# Fine mapping and candidate genes analysis for flesh thickness major locus *Cmqft4.1* of melon (*Cucumis melo* L.)

**DOI:** 10.1093/hr/uhag160

**Published:** 2026-04-17

**Authors:** Shuang Pei, Zicheng Zhu, Hongyu Liu, Xuezheng Wang, Peng Gao, Chunhua Wei, Xian Zhang, Zuyun Dai, Zhongzhou Yang, Xufeng Fang, Shi Liu, Feishi Luan

**Affiliations:** College of Horticulture, Northeast Agricultural University, Harbin, Heilongjiang 150030, China; Key Laboratory of Biology and Genetic Improvement of Horticulture Crops (Northeast Region), Ministry of Agriculture and Rural Affairs, Northeast Agricultural University, Harbin, Heilongjiang 150030, China; College of Horticulture, Northeast Agricultural University, Harbin, Heilongjiang 150030, China; Key Laboratory of Biology and Genetic Improvement of Horticulture Crops (Northeast Region), Ministry of Agriculture and Rural Affairs, Northeast Agricultural University, Harbin, Heilongjiang 150030, China; College of Horticulture, Northeast Agricultural University, Harbin, Heilongjiang 150030, China; Key Laboratory of Biology and Genetic Improvement of Horticulture Crops (Northeast Region), Ministry of Agriculture and Rural Affairs, Northeast Agricultural University, Harbin, Heilongjiang 150030, China; College of Horticulture, Northeast Agricultural University, Harbin, Heilongjiang 150030, China; Key Laboratory of Biology and Genetic Improvement of Horticulture Crops (Northeast Region), Ministry of Agriculture and Rural Affairs, Northeast Agricultural University, Harbin, Heilongjiang 150030, China; College of Horticulture, Northeast Agricultural University, Harbin, Heilongjiang 150030, China; Key Laboratory of Biology and Genetic Improvement of Horticulture Crops (Northeast Region), Ministry of Agriculture and Rural Affairs, Northeast Agricultural University, Harbin, Heilongjiang 150030, China; College of Horticulture, Northwest A&F University, Yangling 712100, China; College of Horticulture, Northwest A&F University, Yangling 712100, China; Anhui Jianghuai Horticulture Technology Co., Ltd., Hefei 230031, China; Anhui Jianghuai Horticulture Technology Co., Ltd., Hefei 230031, China; College of Horticulture, Northeast Agricultural University, Harbin, Heilongjiang 150030, China; Key Laboratory of Biology and Genetic Improvement of Horticulture Crops (Northeast Region), Ministry of Agriculture and Rural Affairs, Northeast Agricultural University, Harbin, Heilongjiang 150030, China; College of Horticulture, Northeast Agricultural University, Harbin, Heilongjiang 150030, China; Key Laboratory of Biology and Genetic Improvement of Horticulture Crops (Northeast Region), Ministry of Agriculture and Rural Affairs, Northeast Agricultural University, Harbin, Heilongjiang 150030, China; College of Horticulture, Northeast Agricultural University, Harbin, Heilongjiang 150030, China; Key Laboratory of Biology and Genetic Improvement of Horticulture Crops (Northeast Region), Ministry of Agriculture and Rural Affairs, Northeast Agricultural University, Harbin, Heilongjiang 150030, China

## Abstract

Flesh thickness (FT) is the edible portion of melon fruit and directly affects its utilization rate, with an important influence on commerciality and yield. In this study, the phenotype of *F*_2_ populations derived from PI 282448 (thin-fleshed, ssp. *melo*, wild type) and M4-135 (thick-fleshed, ssp. *melo*, cultivar) showed that flesh thickness is a quantitative trait. Graded Pool-seq (GPS) combined with genetic linkage analysis identified a stable major-effect quantitative trait locus (*Cmqft4.1*) related to flesh thickness on chromosome 4. Genetic mapping narrowed down the *Cmqft4.1* locus to a 222-kb interval using the near-isogene line NIL_BC_3_*F*_2_ population, which contained 13 candidate genes. Through gene expression and sequence alignment, *MELO3C012896.2* (annotated as Ethylene-responsive transcription factor 1B-like) was predicted as the key candidate gene for flesh thickness in melon. A 286-bp deletion in the promoter region of *MELO3C012896.2* was present in multiple thick-fleshed melon natural materials. These results provide a theoretical basis for further gene functional verification and breeding programs focused on flesh thickness in melon.

## Introduction

Melon (*Cucumis melo* L.) is one of the most important cucurbit crops cultivated worldwide and provides various nutrients [1]. Flesh thickness (FT) is a measure of the edible part of the melon fruit, directly affects its utilization rate, and significantly influences its commercialization and yield. During melon domestication, FT has undergone strong artificial selection. The FT of wild melon is very thin and is not edible, whereas after domestication and improvement, landraces and cultivated varieties display thicker FT compared with the wild type [2, 3], suggesting FT is an important trait in the domestication and improvement of melons. In recent years, multiple genetic studies have mainly focused on the fruit developmental traits of cucurbit crops, including *CsHEC1* for cucumber fruit neck length [4], *CsTRM5*, *CsFUL1*, *CsSF2*, and *CsCRC* for cucumber fruit shape and size [5–8], *CmFSI8*/*CmOFP13* for melon fruit shape [9], *CmCLV3* for melon carpel number [10, 11], and *ClFSI* for watermelon fruit shape [12]. Reports related to melon FT primarily describe quantitative trait locus (QTL) analyses, but the key regulatory genes remain largely unknown. The lack of key genes has limited further research into gene function and molecular regulatory mechanisms for melon FT. Therefore, fine-mapping and cloning key regulatory genes for FT is of significant theoretical value for the molecular breeding of melon.

FT is a typical quantitative trait that is closely associated with fruit developmental processes, and several QTLs associated with FT have been identified in cucurbit crops. In melon, FT-related QTLs *qFT5*, *qFT11*, and *qFT12* [13]; *EPT3.1*, *EPT3.2*, *EPT5.1*, and *EPT6.1* [14]; and *paqt5.1*, *paqt6.1*, *paqt8.1*, *ptqt5.1*, and *ptqt8.1* [3, 15] were identified on chromosomes 3, 5, 6, 8, 11, and 12. Díaz *et al*. [3] further found that *ptqt8.1* was located within a selected region on chromosome 8, which aligns with the QTL region for fruit length and diameter during the domestication process of ssp. *agrestis*. In cucumber, Wang *et al*. [16] identified two QTLs (*mfth3.1*, *mfth5.1*) for FT on chromosomes 3 and 5 with contribution rates of 15.41 and 27.02%, respectively. A major QTL (*fft2.1*) for FT was fine mapped in a 0.19-Mb interval on chromosome 2; *Csa2M058670.1* (Putative histone-lysine N-methyltransferase) was predicted as the candidate gene [17]. *Csa2M058670.1* was the homologous gene of *At2g18850* in *Arabidopsis*, which was found to be involved in cell growth and division [18]. In addition, some QTL loci related to FT have been reported in pumpkin (*qpt8-a*, *qpt8-b*, *qpt9-a*, *fft4.1*, *fft9.1*, *fft14.1*, *fft19.1*, *fth1.1*, *fth4.1*, *fth5.1*, *fth6.1*, *fth14.1*, *fth16.1*) [19–21], papaya (*THI_2016–4*, *THI_2016–9*, *THI_2017–9*) [22] and wax gourd (*ft3.1*, *ft5.1*, *ft9.1*) [23].

In cucurbit crops, most fruit development traits are quantitative traits [24, 25], and fine-mapping key genes was relatively difficult using *F*_2_ populations. The genetic background of near-isogenic line (NIL) populations is relatively consistent, effectively reducing the interference of background differences with target genes [26]. Currently, the strategy of constructing NIL populations using marker-assisted selection (MAS) has successfully cloned key candidate genes for quantitative traits in cucurbit crops, including *Clbl* for watermelon branchless, *FS5.2* for cucumber fruit length, and *CmMt2* for melon mottling rind [27–29].

In this study, the thin-fleshed wild PI 282448 (ssp. *melo*, wild type, *P*_1_) and the thick-fleshed cultivar M4-135 (ssp. *melo*, cultivar, *P*_2_) were crossed to generate *F*_1_, *F*_2_, and backcross populations. A chromosome fragment associated with melon FT was identified using Graded Pool-Seq (GPS) technology. A stable major-effect QTL (*Cmqft4.1*) for FT was detected based on 2 years of *F*_2_ population data. The near-isogenic line (NIL_*Cmqft4.1*) containing *Cmqft4.1* was developed using PI 282448 as the recurrent parent through three consecutive backcrossing (BC) generations, combined with foreground and background MAS. The NIL_*Cmqft4.1* segregated population (BC_3_*F*_2_) was employed for fine mapping of the *Cmqft4.1* locus. Candidate gene *MELO3C012896.2* (annotated as Ethylene-responsive transcription factor 1B-like) for *Cmqft4.1* was predicted through expression pattern analysis combined with alignment of coding and promoter sequences. These findings provide a theoretical basis for the gene function verification and molecular regulatory mechanisms of melon FT.

## Results

### Phenotype characterization and genetic analysis of FT in melon

**Figure 1:**
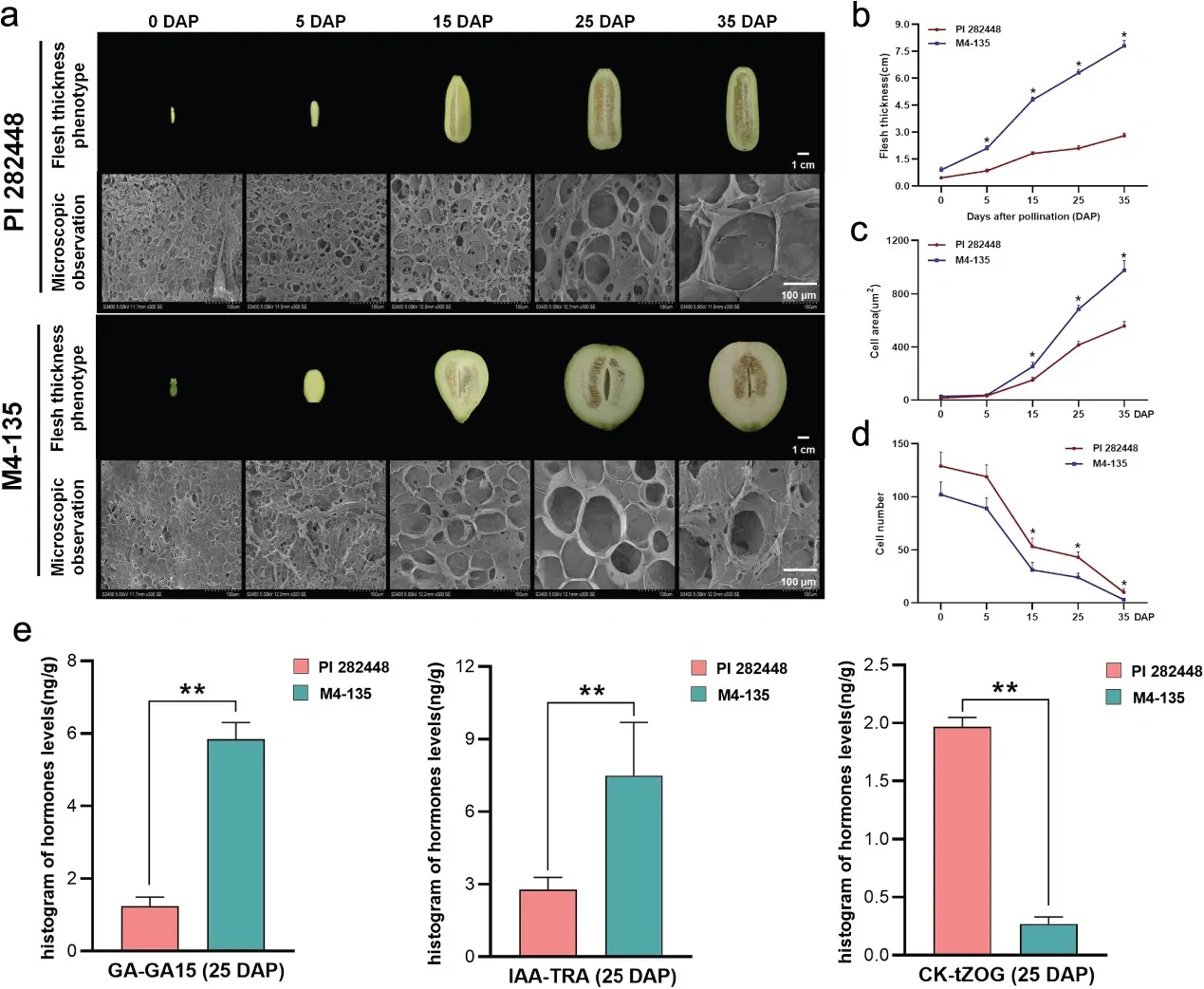
FT phenotypes, microscopic observation, and hormone content of parental lines at different developmental stages. (a) Phenotype and microscopic observation of FT at 0, 5, 15, 25, and 35 DAP in PI 282448 and M4-135. Scale bars for flesh phenotype and microscopy images represent 1 cm and 100 μm, respectively. (b–d) FT determination, single-cell area, and cell numbers in the same area, respectively. ^*^P < 0.05. (e) Hormone content of parental lines at 25 DAP. ^**^P < 0.01.

FT differed significantly between PI 282448 and M4-135 at 5–35 days after pollination (DAP). Flesh cell area and number differed significantly between the parental lines (Fig. 1a). At the fully mature stage (35 DAP), FT of PI 282448 and M4-135 was 2.80 ± 0.15 and 7.70 ± 0.20 cm, respectively (Fig. 1b). The cell area in flesh of PI 282448 was significantly smaller than that in M4-135 (558.00 ± 36.00 vs. 975.00 ± 74.00 μm^2^; Fig. 1c). Moreover, M4-135 exhibited a lower cell number per area compared with PI 282448 at 35 DAP (3.00 ± 1.00 cells / 0.01 mm^2^ vs. 10.00 ± 2.00 cells / 0.01 mm^2^, Fig. 1d), indicating that the FT difference might mainly relate to cell area and number. A total of 17 endogenous hormones were further detected in flesh tissue of parental lines at 25 DAP (Supplementary Data Table S1), with the contents of Gibberellin A15 (GA-GA15) with 1.55 ± 0.59 vs. 5.84 ± 0.56 ng/g, Indole-3-acetic acid tryptamine (IAA-TRA) with 2.85 ± 0.52 vs. 7.50 ± 0.69 ng/g, and Cytokinin-trans-Zeatin-O-glucoside (CK-tZOG) with 1.97 ± 0.08 vs. 0.27 ± 0.06 ng/g exhibiting the most significant differences between PI 282448 and M4-135 (Fig. 1e).

**Figure 2:**
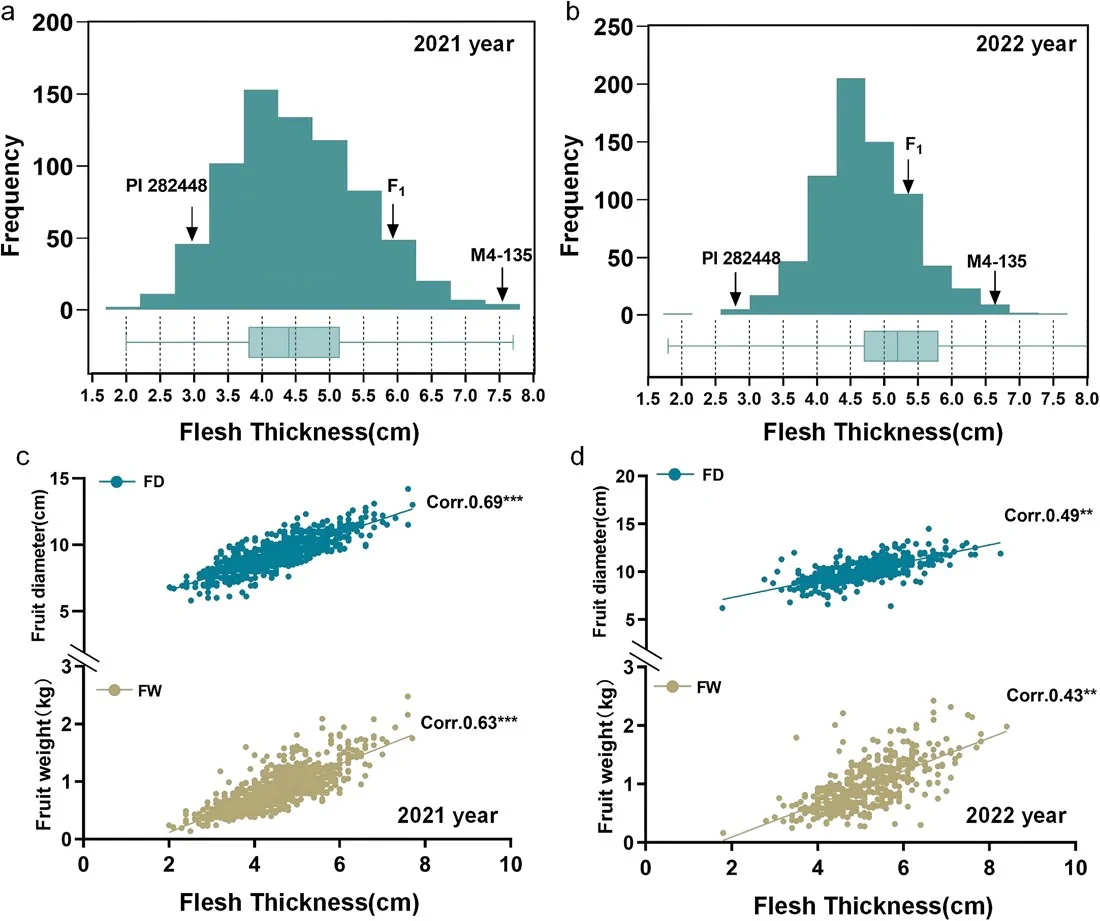
Frequency distributions of FT in 2021 (a) and 2022 (b), and correlation between FD, FW, and FT in 2021 (c) and 2022 (d) in the *F*_2_ population. ^**^P < 0.01, ^***^P < 0.001.

For the *F*_2_ populations, FT ranged from 1.70 to 8.80 cm and 1.80 to 8.40 cm in 2021 and 2022, respectively (Table 1). The FT frequency distributions displayed continuous variation among *F*_2_ individuals across 2 years of data (Fig. 2a and b), indicating that melon FT is a quantitative trait. Furthermore, FT showed a significant positive correlation with fruit diameter (FD) and fruit weight (FW), with a correlation index (*R*^2^) of 0.69 and 0.63 in 2021, and 0.49 and 0.43 in 2022 (Fig. 2c and d). Additionally, the frequency distribution analysis of the *F*_2_ population revealed that five other fruit developmental traits (FD, fruit length [FL], FW, cavity length [CL], and cavity diameter [CD]) were also quantitative traits (Supplementary Data Fig. S1).

**Table 1:** Segregation of *F*_2_ population after crossing PI 282448 and M4-135

Year	Traits	Mean	SD	Range	Kurtosis	Skewness
2021	Flesh thickness (cm)	4.51	0.98	1.70–8.80	0.38	0.32
2022	Flesh thickness (cm)	5.13	0.95	1.80–8.40	0.26	0.32

### Graded Pool-seq, quantitative trait locus analysis and marker-assisted selection verification for flesh thickness

**Figure 3:**
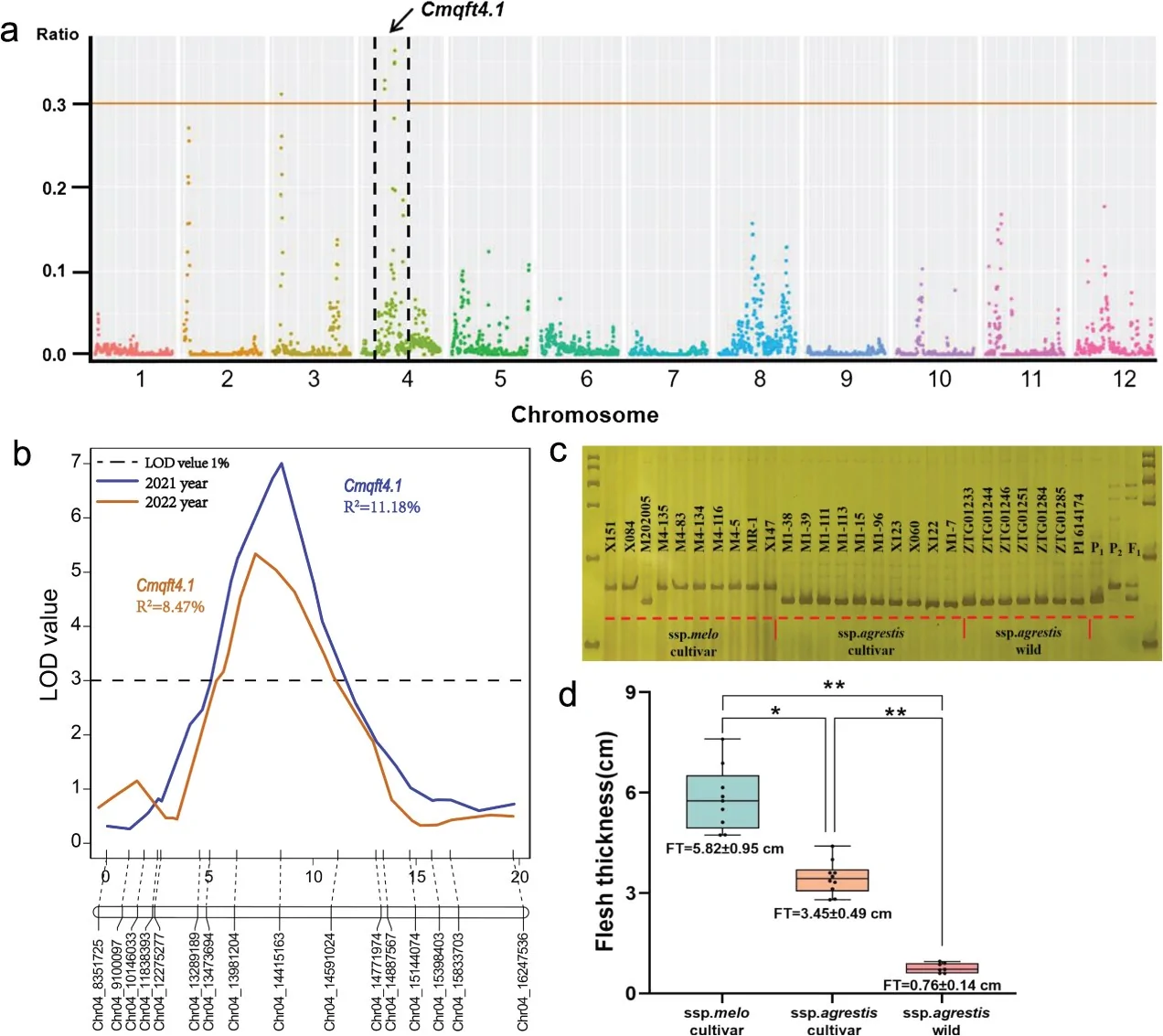
GPS, QTL analysis, and MAS verification of FT. (a) GPS analysis and key region of FT on chromosome 4. (b) QTL initial mapping intervals of FT over 2 years of *F*_2_ population data. (c) Genotyping of InDel marker *Chr04_14415163* in 27 melon accessions with different FT and and of different subspecies. Markers from bottom to top represent fragment sizes of 100, 250, 500, 750, 1000, and 2000 bp, respectively. (d) FT phenotypes of 27 melon accessions.

The three gene pools yielded approximately 24.53, 23.25, and 23.14 Gb clean bases, with a Q30 of 93.86, 93.15, and 93.59%, respectively. PI 282448 and M4-135 yielded approximately 11.66 and 11.68 Gb clean bases, with Q30 of 92.27 and 93.12%. We identified 4 750 793 SNPs and 20 989 insertion/deletion (InDel) loci from the parental lines. After ridit analysis and *P* value calculation, two chromosome segments related to melon FT were detected in a 500-kb region (from 3 400 000 to 3 900 000 bp) on chromosome 3 and a 1.20-Mb region (from 9 500 000 to 10 000 000 bp and 13 700 00 to 14 400 000 bp) on chromosome 4, respectively (Fig. 3a).

Sixteen polymorphic markers in the GPS region were designed and genotyped for *F*_2_ individuals over 2 years (618 plants in 2021 and 413 plants in 2022, respectively, for initial QTL mapping). No significant QTL associated with FT was detected in the corresponding signal region of chromosome 3 (Supplementary Data S2). Only a major effective stable QTL for FT (*Cmqft4.1*) was detected in 1.30 Mb (from 13 289 189 to 14 591 024 bp; Fig. 3b) between two markers *Chr04_13 289 189* and *Chr04_14 591 024* on chromosome 4; the contribution rates (*R*^2^) of *Cmqft4.1* were 11.18% and 8.47% in 2021 and 2022, with LOD values of 7.31 and 5.22 (Table 2). QTLs for FD (*Cmqfd4.1*) and FW (*Cmqfw4.1*) were also detected in the same QTL region of *Cmqft4.1*; the *R*^2^ values were 10.33 and 7.79% for *Cmqfd4.1* and 8.33 and 6.69% for *Cmqfw4.1* in 2021 and 2022, respectively (Supplementary Data Fig. S3; Supplementary Data Table S2).

**Table 2:** QTL analysis of FT in *F*_2_ populations in the years 2021 and 2022

Trait	Year	Population	Chr	Left marker	Right marker	*R* ^2^ (%)	LOD value
FT	2021	*F*_2_ (618)	4	*Chr04_13 289 189*	*Chr04_14 591 024*	11.18	7.31
FT	2022	*F*_2_ (413)	4	*Chr04_13 289 189*	*Chr04_14 591 024*	8.47	5.22

We also genotyped 27 melon accessions with different subspecies and FT using InDel marker *Chr04_14415163* in the QTL mapping region. Nine thick-fleshed lines (ssp. *melo*, cultivar, in addition to M202005) had 156-bp fragments and mean FT 5.82 ± 0.95 cm; in contrast, 17 thin-fleshed lines (10 ssp. *agrestis*, cultivar; 7 ssp. *agrestis*, wild) had 141 bp fragments and mean FT 3.45 ± 0.49 and 0.76 ± 0.14 cm, respectively (Fig. 3c and d). These results suggest that the marker *Chr04_14415163* could distinguish thick-fleshed from thin-fleshed melon accessions.

### Fine mapping of *Cmqft4.1* with NIL_BC_3_*F*_2_ population

Two InDel markers, *Chr04_12275277* and *Chr04_14887567*, in the stable QTL mapping interval were used for foreground selection to identify candidate individuals containing *Cmqft4.1* from each BC generation. Meanwhile, we developed 97 KASP (kompetitive allele-specific PCR) markers to screen for polymorphism between the two parental lines. Among these, 65 polymorphic markers with relatively uniform distribution across the 12 chromosomes of melon were utilized for background selection. The physical distances of all foreground and background markers on the chromosomes are shown in Supplementary Data Fig. S4 and primer sequences for the KASP markers are shown in Supplementary Data Table S3.

The BC_1_*F*_1_ population was generated by crossing *F*_1_ with PI 282448 (the recurrent parent) and a total of 152 heterozygous individuals were selected for background detection at the seedling stage. We subsequently selected individuals with highest proportion of recurrent parent genome (PRPG) for the next generation of backcrossing. For the BC_2_*F*_1_ and BC_3_*F*_1_ populations, the selection steps were consistent with those of the BC_1_*F*_1_ population, with 77 and 91 candidate individuals selected for background detection, respectively. The genotypes of the top 15 high-PRPG individuals on 12 chromosomes across different BC generations are shown in Supplementary Data Fig. S5a-c; the highest PRPG values of the BC_1_*F*_1_, BC_2_*F*_1_ and BC_3_*F*_1_ generations were 89.66, 95.97, and 98.44%, respectively (Supplementary Data Fig. S5d–f).

**Figure 4:**
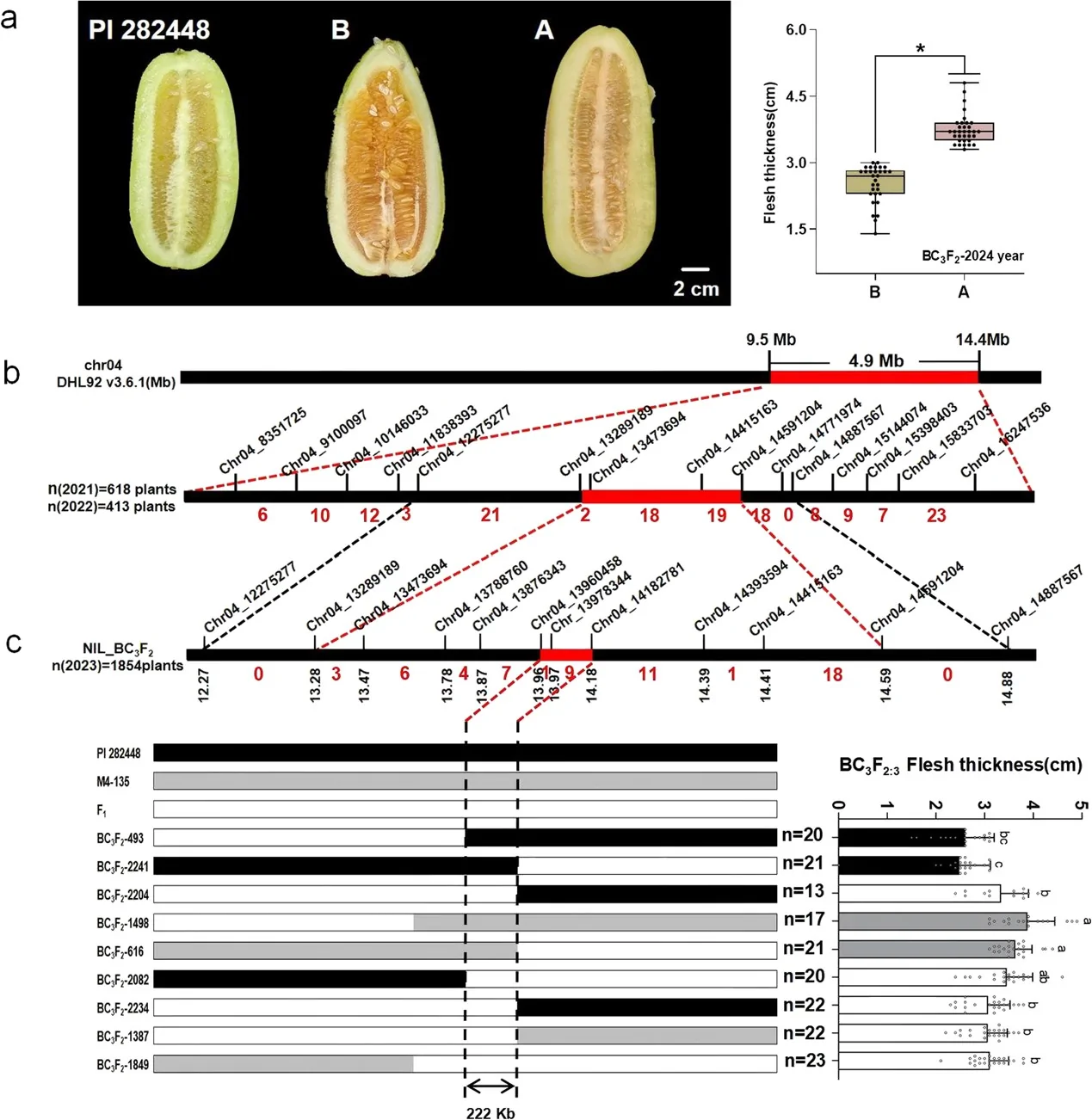
Genetic analysis and fine mapping of FT trait. (a) FT phenotype of different genotypes in NIL_BC_3_*F*_2_ population, Bar = 2 cm, ^*^P < 0.05. (b) Target interval of GPS analysis. (c) Fine mapping interval for FT locus *Cmqft4.1*.

To further narrow the mapping region of *Cmqft4.1*, the NIL_BC_3_*F*_2_ segregated population of *Cmqft4.1* was obtained after self-crossing 10 high-PRPG individuals from the BC_3_*F*_1_ generation for gene fine mapping. We first analysed the inheritance of *Cmqft4.1* using 138 BC_3_*F*_2_ plants; 35, 70, and 33 plants were homozygous B (genotypes as in PI 282448) with FT of 1.40–3.00 cm, heterozygous H with FT of 3.00–3.90 cm, and homozygous A (genotypes as in M4-135) with FT of 3.30–4.80 cm, respectively, fitting a 1:2:1 ratio (*χ*^2^ = 0.165, *P* = 0.861; Table 3), FT was significantly difference between the individuals with genotype B and A (2.52 ± 0.43 vs. 3.75 ± 0.34 cm; Fig. 4a). To narrow down the *Cmqft4.1* interval, *Chr04_13 289 189* and *Chr04_14 591 024* were used for genotyping 1854 BC_3_*F*_2_ plants and 83 recombinants were identified. Eight polymorphic markers were designed in the 1.30-Mb region to genotype recombinants (Fig. 4c). All recombinants were self-pollinated to obtain BC_3_*F*_2:3_ families, and at least 25 plants of each BC_3_*F*_2:3_ family were planted to observe the FT phenotype for gene fine mapping. The *Cmqft4.1* locus was finally located in a 222-kb region between the two markers *Chr04_13960458* and *Chr04_14182781* (Fig. 4c), containing 13 candidate genes; 10 of them (Supplementary Data Table S4) had non-synonymous SNP sites in coding sequences, *cis*-acting element mutations in promoter sequences, or structure variations between the parental lines.

**Table 3:** Segregation of FT in BC_3_*F*_2_ population

Generation	Homozygous genotype (B)	Heterozygous genotype (H)	Homozygous genotype (A)	Actual ratio	*χ* ^2^	*P* value
BC_3_*F*_2_	35	70	33	1:2:1	0.165	0.861

### Candidate gene selection and sequence analysis

**Figure 5:**
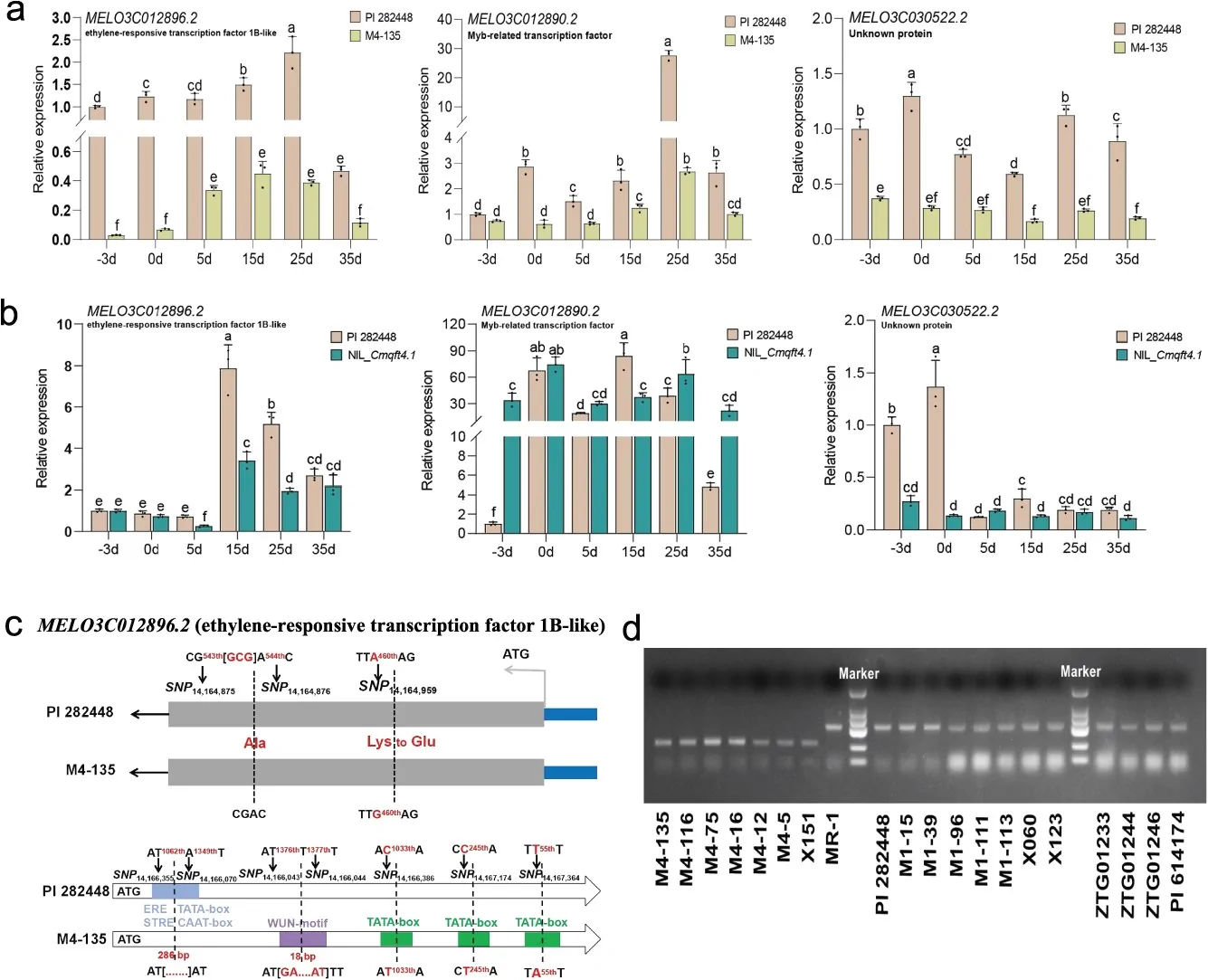
Candidate gene prediction. Expression analysis of three candidate genes in parental lines (a) and between PI 282448 and NIL_*Cmqft4.1* (b) during six flesh development stages. (c) Sequence alignment of coding and promoter region of *MELO3C012896.2* in parental lines. (d) Genotype of 286-bp deletion in *MELO3C012896.2* in 20 melon accessions. Markers from bottom to top represent fragment sizes of 100, 250, 500, 750, 1000, and 2000 bp, respectively.

We then analysed the expression patterns of 10 candidate genes in flesh tissues at six different developmental stages between two parental lines. The expression levels of *MELO3C012896.2*, *MELO3C012890.2*, and *MELO3C030522.2* were significantly different at the six developmental stages between PI 282448 and M4-135 (Fig. 5a). In contrast, no consistent or significant differential expression trends were observed for the remaining seven candidate genes between the two parental lines during fruit developmental stages (Supplementary Data Fig. S6). We therefore hypothesize that these seven genes have a relatively low correlation with the melon FT trait. To eliminate the effects of genetic background differences on the expression of candidate genes, we further analysed the expression level of candidate genes for FT at six developmental stages in PI 282448 and NIL_*Cmqft4.1*. The phenotype of FT between PI 282448 and *NIL_Cmqft4.1* showed a significant difference at 15–35 DAP (Supplementary Data Fig. S7). The expression pattern analysis found that *MELO3C012896.2* was the only candidate gene consistent with the developmental process of FT, exhibiting significantly higher expression levels in PI 282448 compared with NIL_*Cmqft4.1* at 5–25 DAP (Fig. 5b). However, the expression levels of the other nine candidate genes showed no significant differences during the critical developmental period between PI 282448 and the *NIL_Cmqft4.1* line (Fig. 5b and Supplementary Data Fig. S8).

The coding and promoter sequences of *MELO3C012896.2* were compared between the parental lines according to cloning results. For the coding region, *MELO3C012896.2* had one non-synonymous SNP_14 164 959_ (G → A, Lys → Glu); a 3-bp insertion at the SNP_14 164 875_ position forms a new amino acid, alanine (Ala), in PI 282448. For promoter sequences, three SNP mutations, SNP_14 167 364_, SNP_14 167 174_, and SNP_14 166 386_ (T → A, C → T, C → T), result in a change in the *cis*-acting element of the TATA-box, an 18-bp insertion at the SNP_14 16 043_ position forms a new *cis*-acting element WUN motif, and the sequence of a 286-bp deletion at the SNP_14 166 355_ position contained ERE, STRE, TATA-box, and CAAT-box, four *cis*-acting elements, in M4-135 (Fig. 5c). We then analysed SNP mutation sites of *MELO3C012896.2* using the re-sequencing data of 73 natural melon lines. SNP_14 164 959_ consistently presented as A, as in PI 282448 and 16 thin-fleshed melon accessions, and as G, as in M4-135, in 57 thick-fleshed accessions, which was associated with significant differences in FT (1.35 ± 0.56 4.30 ± 1.37 cm). The genotype of SNP_14 164 959_ (G → A) co-segregated with the FT phenotype of the 73 natural melon lines (Supplementary Data Fig. S9). In addition, we further detected a 286-bp nucleotide variant of *MELO3C012896.2* among 20 natural melon accessions with different FT, and PCR amplification fragments of 291 bp, as in M4-135 and the six thick-fleshed melon lines (in addition to MR-1), while PI 282448 and 11 thin-fleshed melon lines displayed fragments of 577 bp. This 286-bp polymorphism demonstrated stability within the natural germplasm and co-segregated with thin and thick flesh of melon (Fig. 5d).

**Figure 6:**
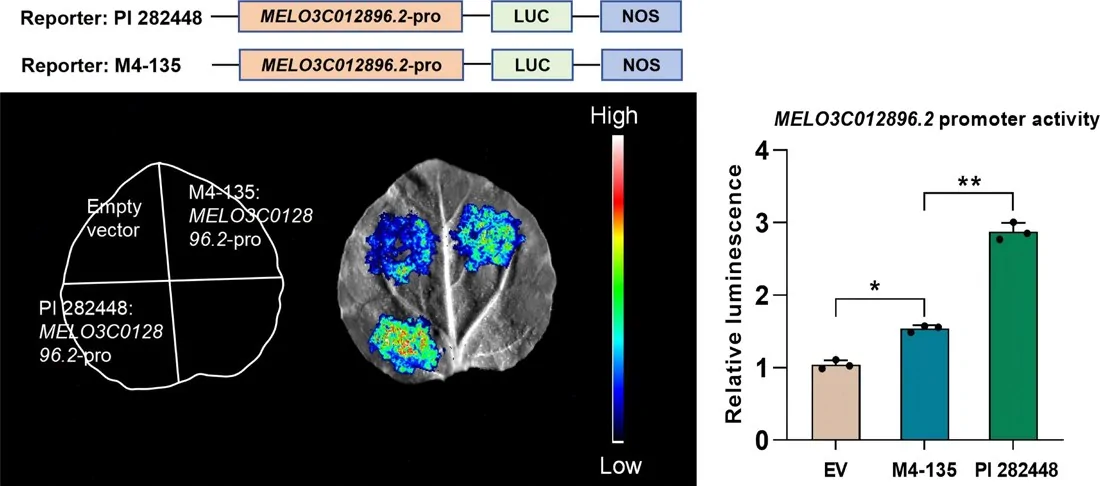
Promoter activity analysis of *MELO3C012896.2* between two parental lines.

To further investigate whether the 286-bp structural variation affects *MELO3C012896.2* promoter activity, the *MELO3C012896.2* promoters from two parental lines were successfully constructed into the pGreenII-0800-Luc vector. The fluorescence signal of *Nicotiana benthamiana* leaves containing *MELO3C012896.2*-pro from PI 282448 was stronger than that of M4-135, and the dual-luciferase reporter assay showed that the promoter activity of PI 282448 was significantly increased compared with M4-135 (empty vector [EV] as control; Fig. 6). These results indicate that promoter activity may be a potential factor in the differential expression of *MELO3C012896.2* in FT between the parental lines.

## Discussion

Organ size in plant development is mainly determined by cell expansion and cell proliferation [30–32], including cell expansion duration or rate, cell number, and proliferation arrest time [33, 34]. In this study, scanning electron microscopy (SEM) observation revealed significant differences in flesh cell size between two parental liens at 15–35 DAP (Fig. 1a). PI 282448 exhibited a lower cell area and larger cell numbers within the same areas compared with M4-135 (Fig. 1b and c), and we speculate that cell size may be related to FT development in melon. Similarly, in *Arabidopsis*, an increase in petal cell area also corresponds to a decrease in cell numbers within the same region [35]. However, SEM has technical limitations for observing melon flesh cells, as the tissue structure may be compromised during sample preparation, resulting in significant errors in the calculation of cell area and number. Thus, further observation using paraffin sections is needed to investigate the dynamic processes of cell proliferation and expansion at different developmental stages in flesh tissue of melon.

The FT of melon is a typical quantitative trait regulated by multiple genes; previous research has identified several QTLs related to FT on different chromosomes in melon [3, 13, 36]. However, the regulatory genes remain unknown. In this work, we identified a major stable QTL for FT, *Cmqft4.1*, using a forward genetic approach, predicting that *MELO3C012896.2* is the most potential candidate gene. Evidence supporting the role of *Cmqft4.1* in regulating flesh development is mainly reflected in three aspects.

Firstly, *Cmqft4.1* is a new locus that has not been reported and was located at a stable interval (~1.30 Mb) on chromosome 4 with 2 years of data (Fig. 3b). MAS of FT confirmed that an InDel marker, *Chr04_14415163*, could distinguish thick-fleshed from thin-fleshed melon accessions (Fig. 3c), providing additional support for the QTL within the initial mapping region containing the *Cmqft4.1* locus. Furthermore, we developed an NIL for the *Cmqft4.1* locus, which has greatly promoted the reliability of phenotypic identification and gene fine mapping of the FT trait (Fig. 4). Currently, NIL populations are also widely used for genetic studies of quantitative traits in some horticultural crops [37–39]. Secondly, the significantly higher expression of *MELO3C012896.2* in the thin-fleshed parent PI 282448 at 5–25 DAP (Fig. 5b) corresponds to a critical stage in FT development, indicating that *MELO3C012896.2* may act as a negative regulator of flesh thickening. Thirdly, gene cloning revealed a 286-bp deletion in the promoter of *MELO3C012896.2* in M4-135. Notably, this deletion existed universally in all thick-fleshed melon accessions (Fig. 5d), suggesting a potential selective advantage during domestication. In addition, previous studies showed that insertions and deletions in the promoter region can affect gene function by disrupting *cis*-regulatory elements [40–42]. For instance, a 60-bp deletion in the promoter of *WRKY34* was shown to remove a GATA *cis*-acting element that binds to SWIBs and GATA29, thereby suppressing the expression and transcriptional level of *WRKY34*, ultimately reducing the cold tolerance of cultivated tomato [43]. Similarly, a 229-bp deletion in the *LrAN1b* promoter region was shown to remove three MYB-binding elements and one bHLH-binding element, which impairs the expression of *LrAN1b* and further causes the obstruction of anthocyanin biosynthesis in goji berry [44]. In our work, we identified a 286-bp deletion in the thick-fleshed parent M4-135, which includes four *cis*-acting elements: ERE, STRE, TATA-box, and CAAT-box. Due to the lower expression level and reduced promoter activity of *MELO3C012896.2* in M4-135, we speculated that sequence deletion in the upstream region of *MELO3C012896.2* may potentially influence its transcriptional regulation. However, whether and how this deletion affects transcription factor binding and subsequently cell proliferation or expansion (resulting in fewer but larger cells), thereby contributing to FT formation, remains unclear and requires further gene functional validation through CRISPR/Cas9 knockout technology.

The candidate gene *MELO3C012896.2* encodes Ethylene-responsive transcription factor 1B-like. Ethylene-responsive factors (ERFs) are important transcription factors (TFs) in plants, being involved in fruit development, internode elongation, floral organ formation, and seed germination in the ethylene signaling pathway [45–48]. Previous studies also reported ERF functions in some crops, including banana, kiwifruit, cucumber, and *Arabidopsis*. *MaERF11* regulates fruit ripening of banana [49], while *AeAP2*/*ERF061* and *AeAP2*/*ERF067* are involved in flowering and flower tissue formation of kiwifruit [50], *CsERF025* regulates fruit shape of cucumber [51], and *AtERF38* regulates seed development of *Arabidopsis* [52]. In addition, ERFs are also responsive to the biosynthesis of hormones, including gibberellin, cytokinin, ethylene, auxin, and jasmonate, through feedback regulation, thereby affecting plant and fruit growth and development [53]. In this study, the expression trend of *MELO3C012896.2* was consistent with the FT developmental process, and the contents of GA15, IAA-TRP, and CK-tZOG were significantly different in flesh tissues of parental lines at 25 DAP (Fig. 1e). We therefore speculate that *MELO3C012896.2* affects melon FT by responding to the synthesis of multiple hormones. Similar results have also occurred in other studies; *SNORKEL1* and *SNORKEL2* may interact directly or indirectly with gibberellins through signaling, to induce or inhibit internode elongation in rice [54]. *CsERF69* and *CsERF84* are involved in ovary development in cucumber through cytokinin induction [55]. Although our study indicates that *MELO3C012896.2* may regulate the formation of FT in melon, the hormone content was only determined in flesh sampled from parental lines at 25 DAP. The available data do not reflect the dynamic accumulation of hormones during flesh development, and the correlation between hormone levels and FT variations is not strong. Subsequent research will measure various hormone levels over the whole flesh development period. Moreover, the functional verification of *MELO3C012896.2* should be further investigated to clarify whether this gene is involved in FT formation in melon.

## Materials and methods

### Plant materials and phenotype evaluation

Thin-fleshed PI 282448 (ssp. *melo*, wild type, *P*_1_) and thick-fleshed M4-135 (ssp. *melo*, cultivar, *P*_2_) were used as the parental lines to obtain *F*_1_ and *F*_2_ populations. NILs for the FT locus *Cmqft4.1* were constructed through three consecutive backcrossings using PI 282448 as the recurrent parent. All plants were planted in a greenhouse at the Xiangyang Experimental Agricultural Station of Northeast Agricultural University (Heilongjiang Province, Harbin, China). The *P*_1_, *P*_2_, *F*_1_ (15 plants each), and *F*_2_ populations (618 and 413 plants) were planted in 2021 and 2022 for GPS and QTL analysis. The BC populations BC_1_*F*_1_ (152 plants), BC_2_*F*_1_ (218 plants), and BC_3_*F*_1_ (118 plants) were planted for NIL_*Cmqft4.1* line construction, and the NIL_BC_3_*F*_2_ (*n* = 1854 plants) population was planted in 2023 for recombinant event selection and gene fine mapping.

In addition, 27 melon natural accessions were planted in 2023 for MAS verification of the FT trait (Supplementary Data Table S5). Mature fruits were cut lengthwise and photographed; FT, FD, FL, CL, and CD were measured with a ruler (in centimeters). FW was measured with electronic scales (in kilograms).

### Microscopic observation and endogenous hormone determination of flesh tissue

Flesh samples from two parental lines were collected at different development stages (0, 5, 15, 25, and 35 DAP), cut into 2.0–4.0 mm portions and immersed immediately in 2.5% glutaraldehyde solution. After discarding fixative solution, Samples were washed three times with 0.1 M phosphate buffer (pH = 7.2) for 10 min each. Subsequently, dehydration was performed using sequential ethanol solutions of 50, 70, 90, and 100%, with each step repeated three times for 15 min. The sample were finally transferred to 100% *tert*-butanol solution for 15 min. Observations and photographs of sections were obtained using a scanning electron microscope (S-3400N, Hitachi, Tokyo, Japan). Flesh cell sizes and numbers were calculated using ImageJ software [56].

Flesh samples of PI 282448 and M4-135 at 25 DAP were frozen and ground into powder. Samples of 50 mg were dissolved in 1 mL methanol/water/formic acid (15:4:1, V/V/V), and 10 μL of 100 ng mL^−1^ internal standard mixed solution was added to the extract for quantitation, followed by vortexing, centrifugation, supernatant collection, and concentration. The concentrated samples were then dissolved in 100 μL 80% methanol (V/V), and filtered through a 0.22-μm membrane filter for further liquid chromatography–tandem mass spectrometry (LC–MS/MS) analysis [57, 58]. A total of 75 hormone standards were used for the qualitative and quantitative analysis of endogenous flesh hormones.

### Graded Pool-seq, quantitative trait locus analysis and marker-assisted selection system verification

Fifty thin-fleshed (2.0–3.1 cm), 50 middle-type fleshed (4.1–4.6 cm), and 50 thick-fleshed (6.0–7.7 cm) *F*_2_ individuals were selected for DNA extraction to establish three flesh gene pools. DNA samples from parental lines and three gene pools were re-sequenced with 50× coverage using the Novaseq 6000 platform at the Lianchuan Biological Technology Company (Hangzhou, China). The re-sequenced data were aligned against the melon reference genome DHL92 v3.6.1 (http://cucurbitgenomics.org/ftp/genome/melon/DHL92/v3.6.1/). Single-nucleotide polymorphism (SNP) and InDel sites were detected using Burrows–Wheeler Aligner (BWA) software [59, 60]. After the filtering process, ridit analysis was performed to calculate the *P* value of each variant. The chromosome regions associated with FT were obtained by denoising the *P* value.

According to GPS analysis data, InDel and cleaved amplified polymorphic sequence (CAPS) markers were designed using Primer Premier v5.0 software. InDel markers were devised from sites with at least a 5-bp insertion or deletion between two parental lines. PCR products were separated by 8% denaturing polyacrylamide gel electrophoresis with silver staining. CAPS markers were devised using five restriction enzymes (BsaHI, MboI, HincII, DraI, HindIII) with SNP2CAPS software [61]. PCR products and enzymatic digestion products were detected by 1% agarose gel electrophoresis. Codominant polymorphism markers from parental lines and *F*_1_ generations were selected for *F*_2_ population genotyping. JoinMap v4.0 software (https://www.kyazma.nl/index.php/JoinMap) was used to construct genetic linkage maps and R/qtl v1.5 software (https://rqtl.org/index.html) was used for QTL initial interval analysis.

In addition, we investigated the correlations between genotypes and FT phenotypes using genomic DNA from 27 melon accessions (including 10 ssp. *melo* cultivars; 10 ssp. *agrestis* cultivars; 7 ssp. *agrestis* wild types; Supplementary Data Table S1). InDel marker *Chr04_14415163*, with the highest LOD value in the QTL mapping region, was selected for MAS system verification.

### Construction of NIL_*Cmqft4.1* segregated population and gene fine mapping

Two InDel markers, *Chr04_12275277* and *Chr04_14887567*, located at the boundary of the initial QTL region were used to screen heterozygous individuals for foreground selection from BC_1_*F*_1_ to BC_3_*F*_1_ populations. Additionally, 65 polymorphic KASP markers were distributed across the 12 chromosomes for background selection. Individuals exhibiting a high PRPG were selected for backcrossing with the recurrent parent PI 282448 in each BC generation. Finally, the 10 highest-PRPG individuals from BC_3_*F*_1_ were self-crossed to obtain the BC_3_*F*_2_ population harboring *Cmqft4.1* for subsequent inheritance analysis and gene fine mapping.

PRPGs of all individuals in the BC population were calculated according to Hospital *et al*. [62]. The calculation formula was as follows: PRPG(g) = (*X*(g) + *L*)/2*L*, where PRPG(g), *X*(g), and *L* represent PRPG, the same marker number between the BC population and the recurrent parent, and the total marker number (65 KASP) from the melon genome for background selection, respectively. The distributions of all markers on the 12 chromosomes were determined using MapChart v2.3 software (https://mapchart.software.informer.com), and the PRPG(g) of all backcrossing individuals was analysed using Graphical Genotype GGT v2.0 software (https://ggt.software.informer.com).

Recombinant plants were screened from BC_3_*F*_2_ population (1854 plants) with two markers flanking the initial region of *Cmqft4.1*, and self-pollinated to obtain BC_3_*F*_2:3_ families. The genotypes of recombinants were determined according to FT segregation in the BC_3_*F*_2:3_ families (at least 20 plants for each family). New markers in the QTL mapping region were developed to genotype the recombinant events for fine mapping.

### Candidate gene prediction

The coding regions and promoters of candidate genes in fine mapping region were aligned with parental line re-sequencing data. The coding sequences were compared using the Integrative Genomics Viewer software (http://software.broadinstitute.org/software/igv), and *cis*-acting elements of the promoters were predicted using the online PlantCARE software (http://bioinformatics.psb.ugent.be/webtools/plantcare/html). Candidate genes with non-synonymous SNP sites in the coding sequences or mutations causing *cis*-acting elements in promoters (or structure variations) were chosen for gene expression pattern analysis.

PI 282448, M4-135, and NIL_*Cmqft4.1* flesh tissue was collected at −3, 0, 5, 15, 25, and 35 DAP for RNA extraction. Primers were designed to avoid conserved domains using the National Center for Biotechnology Information (NCBI) database (http://www.ncbi.nlm.nih.gov/Structure/cdd/wrpsb.cgi). A spin column plant total RNA purification kit (Sangon, Shanghai, China) was used to extract flesh RNA, HiScript III RT SuperMix for qPCR+gDNA wiper (Vazyme, Nanjing, China) was used for cDNA synthesis, *MELO3C008032.2* was used as actin from the cucurbit database melon genome (DHL92 v3.6.1), and the 2^− ΔΔCT^ method was used to calculate the relative gene expression level [63].

### Sequence alignment of *MELO3C012896.2*

The coding region and promoter sequence of *MELO3C012896.2* was cloned from two parental lines. PCR products were amplified with KOD One Master Mix (Toyobo, Japan), PCR fragments were purified with a FastPure Gel DNA Extraction Mini Kit (Vazyme, Nanjing, China), and were then linked to the pMD18-T vector (Takara, Japan) and sent to Sangon Biotech (Shanghai, China) for sequencing. DNAMAN 6.0 software (Lynnon Biosoft, USA) was used to compare the coding and promoter sequencing results.

### Promoter activity analysis of *MELO3C012896.2*

The promoter fragments of *MELO3C012896.2* from two parental lines were constructed on the pGreenII-0800-Luc vector and transformed into *Escherichia coli* DH5α cells (Code G6016, AngYu Biotech, Shanghai) for sequencing. The recombinant plasmid with qualified sequencing results was extracted using E.Z.N.A.^®^ Plasmid Mini Kit I (Code D69430, Omega) and transformed into *A. tumefaciens* GV310-pSoupl (Code G6039–2, AngYu Biotech, Shanghai), and the reporter was injected into fresh *N. benthamiana* leaves. After 48–72 h of incubation under low-light conditions, leaf fluorescence signals and relative luciferase (LUC) activity were detected using the fully automated Tanon 5200 chemiluminescence imaging system (Tanon, China) and the Dual-Luciferase Reporter Assay System (Promega, USA), respectively.

### Statistical analysis

Significant differences were analysed using one-way ANOVA with SPSS v25.0 software (SPSS Inc., Chicago, IL, USA). GraphPad Prism v8.0 software (GraphPad Inc., La Jolla, CA, USA) was used to determine frequency distributions, perform correlation analysis, and draw all figures.

## Supplementary Material

Web_Material_uhag160

## Data Availability

The data supporting this article are available within the manuscript and its supplementary materials.
