# Supplementary material for: Fine mapping and candidate genes analysis for flesh thickness major locus Cmqft4.1 of melon (Cucumis melo L.)
Source: Hortic Res. 2026 Apr 17;13(9):uhag160. doi: 10.1093/hr/uhag160 (PMC13532403; doi:10.1093/hr/uhag160)
Supplement: Supplementary file 1 [file web_material_uhag160.zip › Supplemental Figures (Revision).pdf]

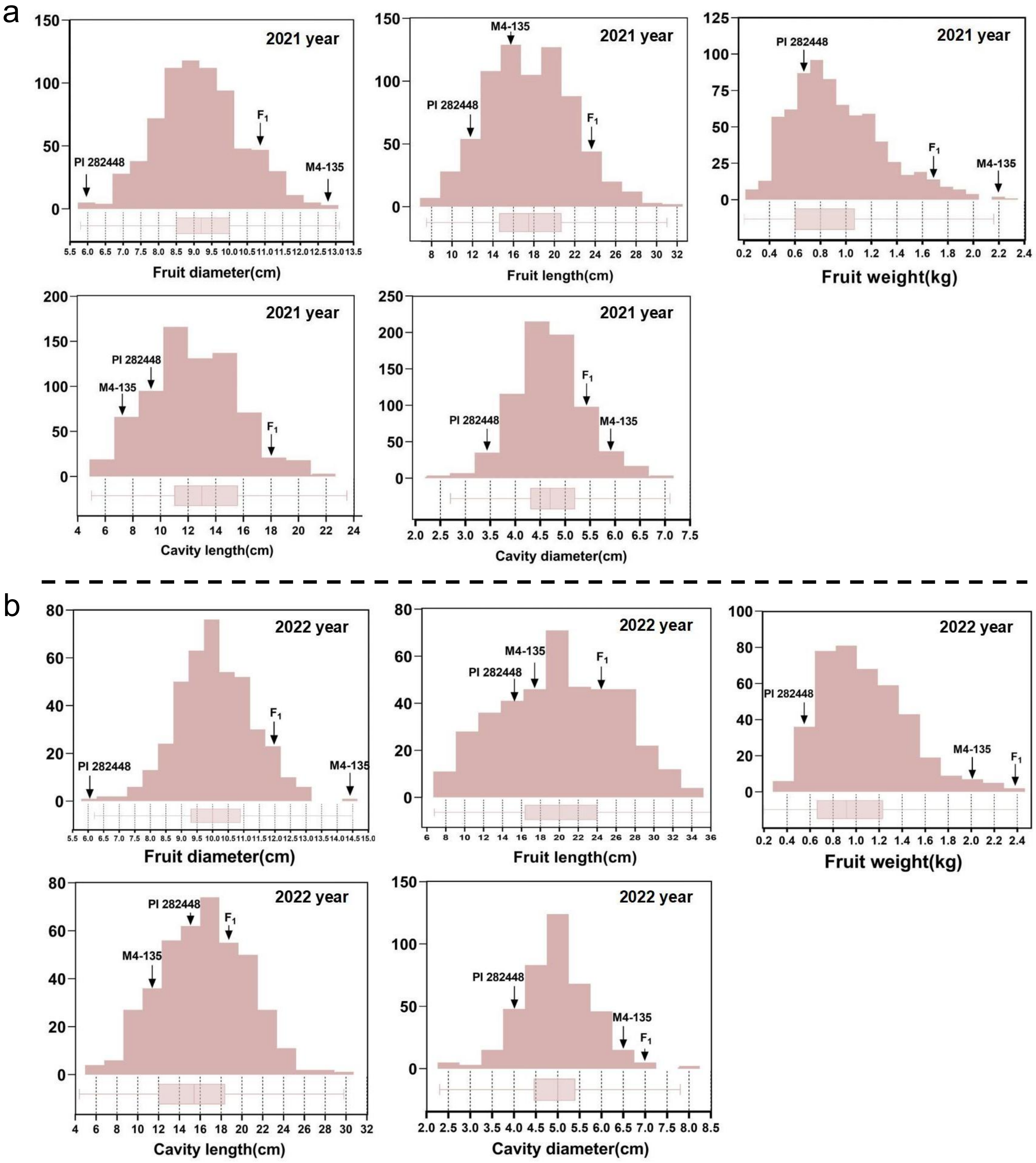
a
2021 year
PI 282448
F1
M4-135
Fruit diameter(cm)
Fruit length(cm)
Fruit weight(kg)
Cavity length(cm)
Cavity diameter(cm)
b
2022 year
PI 282448
F1
M4-135
Fruit diameter(cm)
Fruit length(cm)
Fruit weight(kg)
Cavity length(cm)
Cavity diameter(cm)

Figure S1.

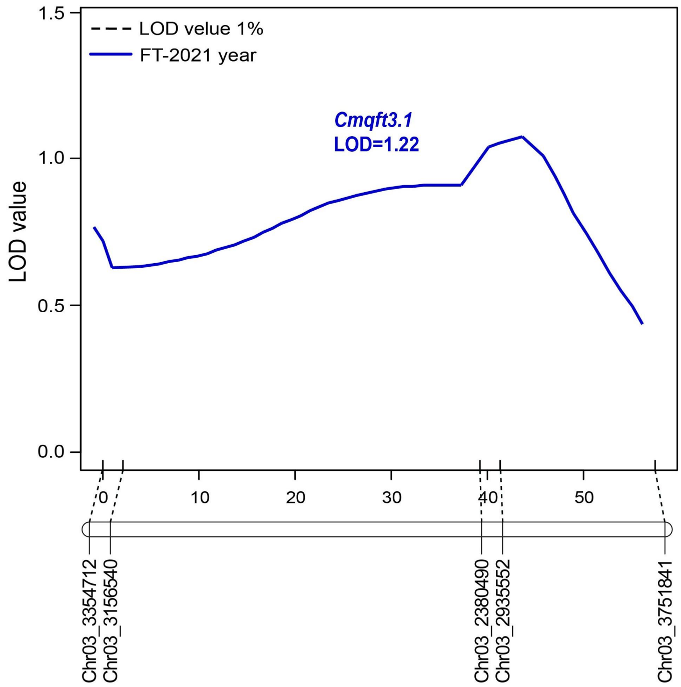

Figure S2.

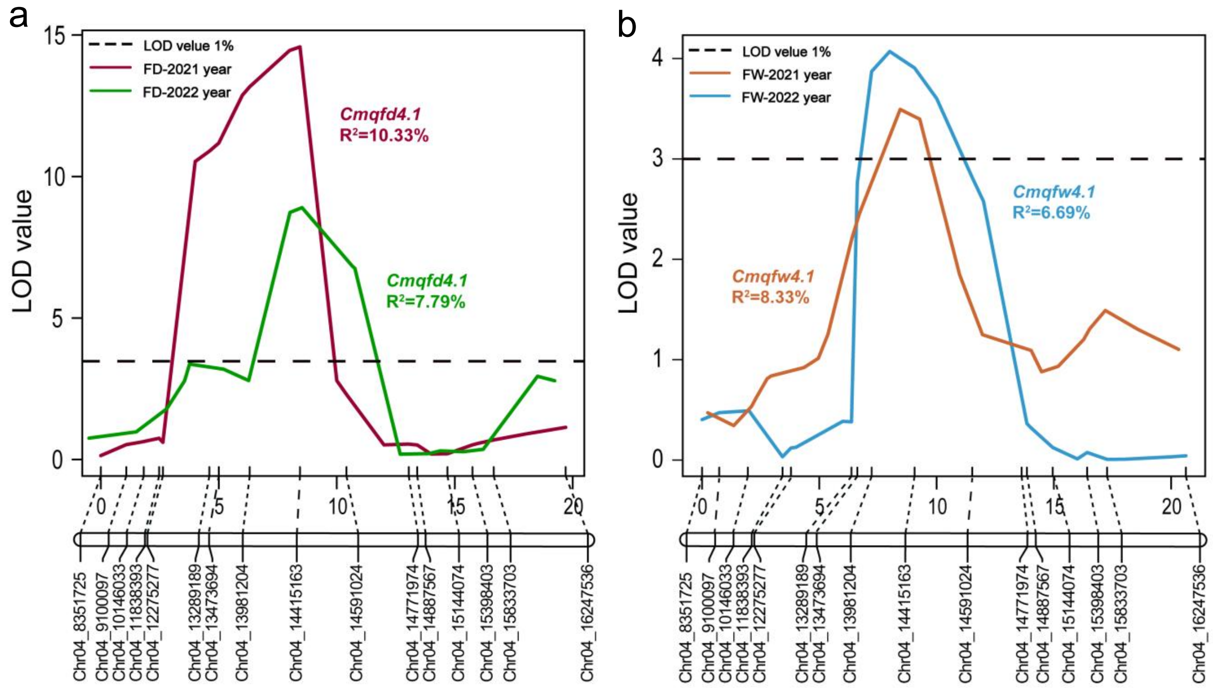

Figure S3.

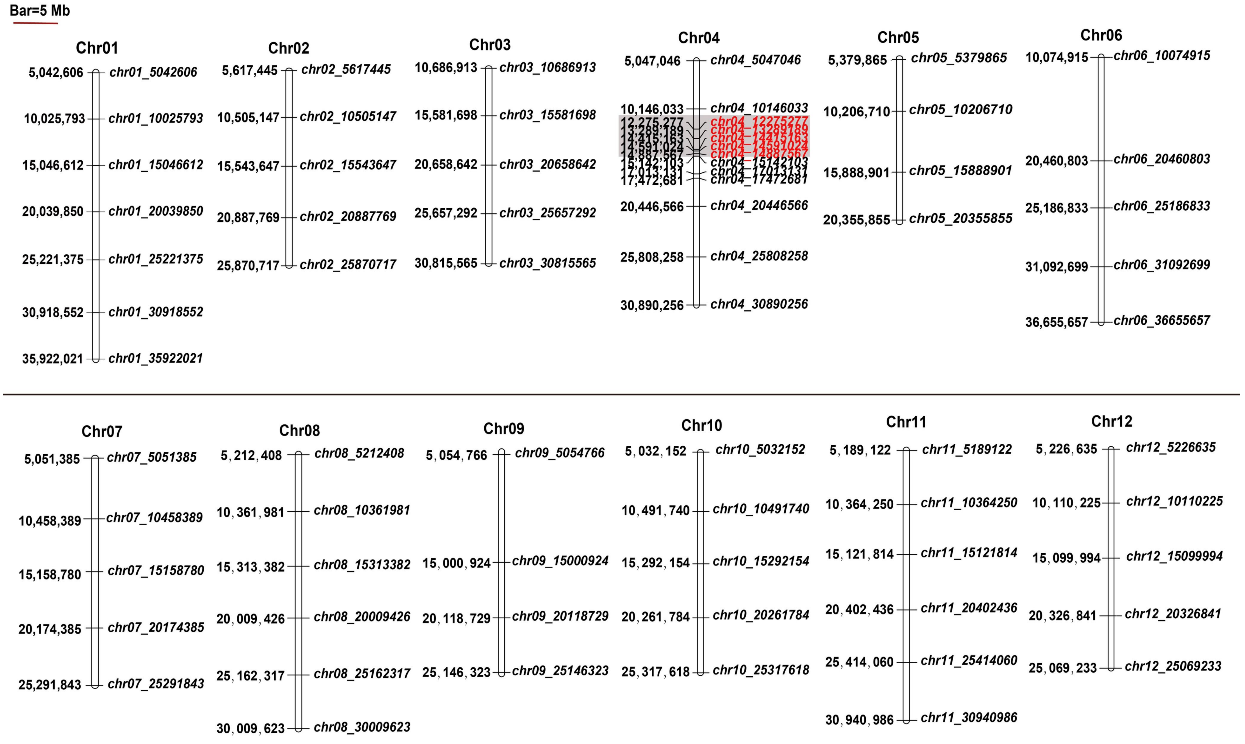

Figure S4.

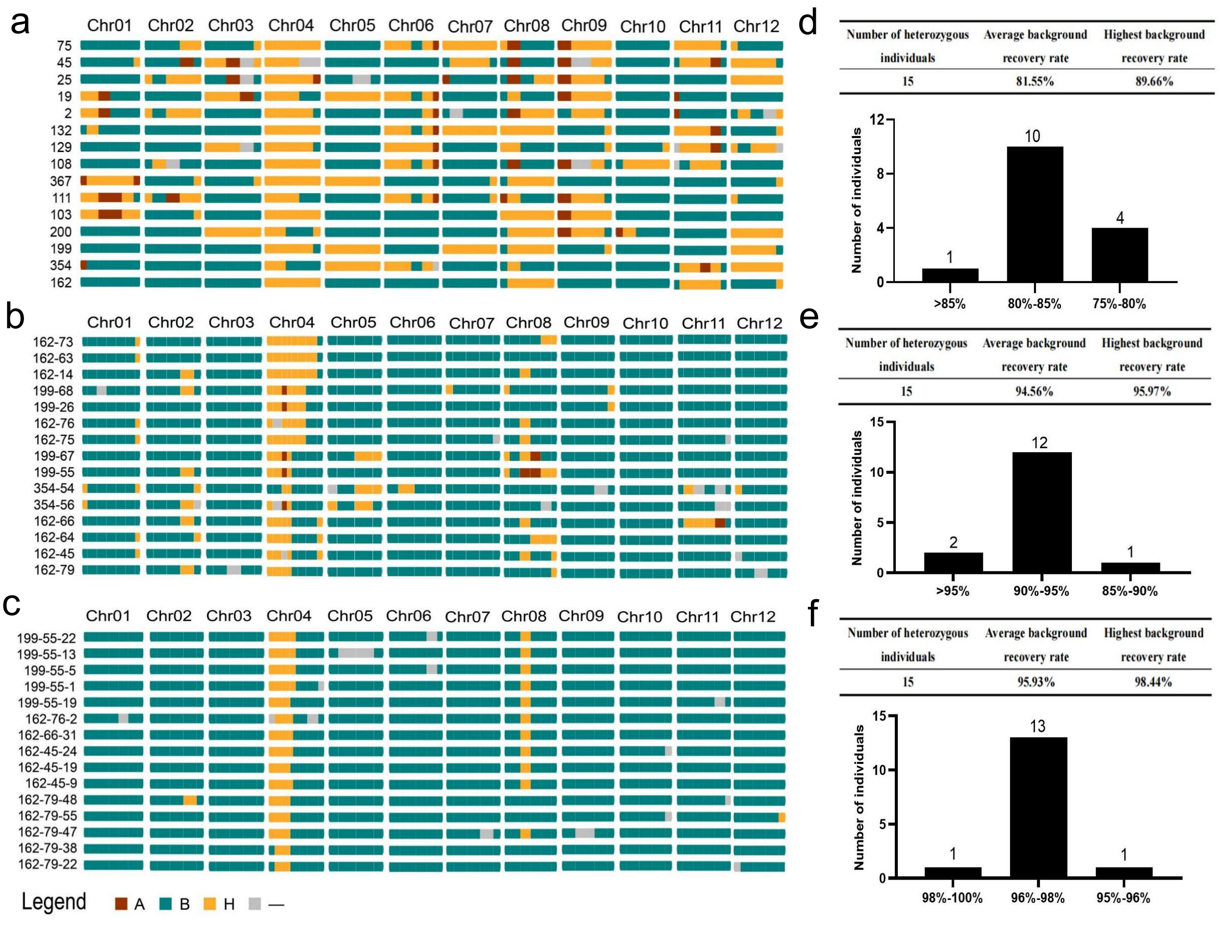

Figure S5.

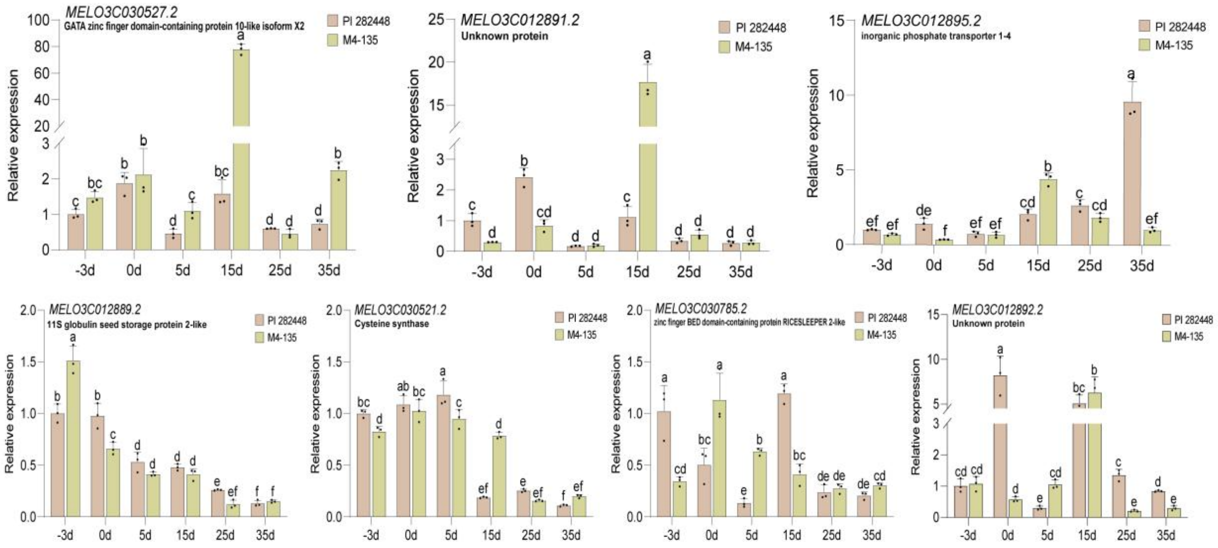

Figure S6.

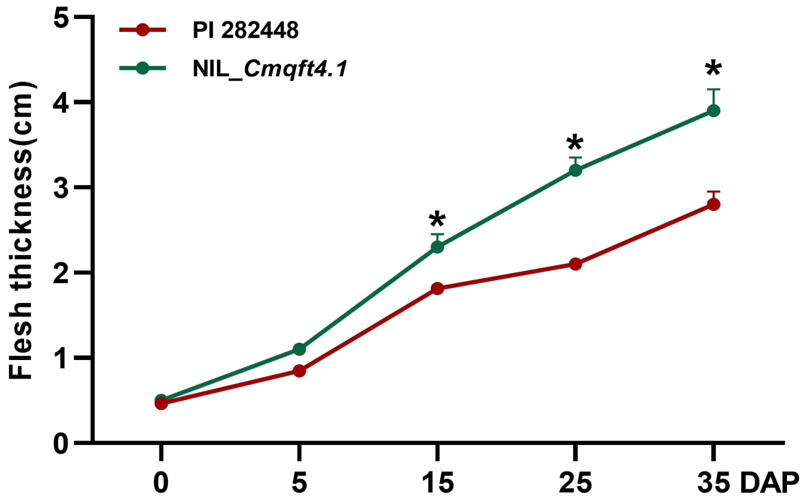

Figure S7.

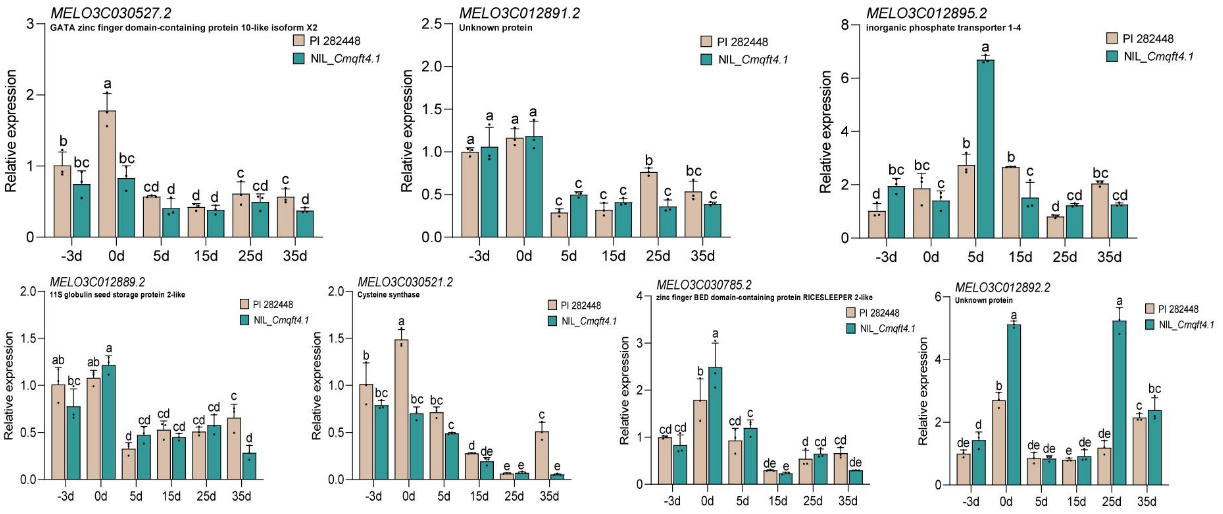

Figure S8.

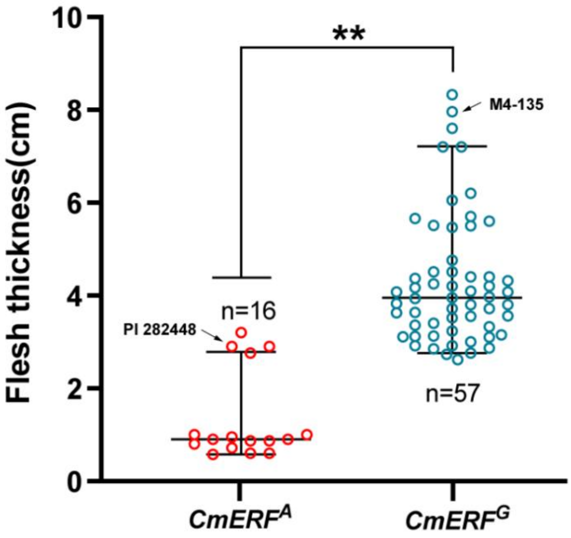

Figure S9.
