# Supplementary material for: Fine mapping and candidate genes analysis for flesh thickness major locus Cmqft4.1 of melon (Cucumis melo L.)
Source: Hortic Res. 2026 Apr 17;13(9):uhag160. doi: 10.1093/hr/uhag160 (PMC13532403; doi:10.1093/hr/uhag160)
Supplement: Supplementary file 1 [file web_material_uhag160.zip › Supplemental Tables (Revision).doc]

**Table S1** Endogenous hormones analysis of 25 DAP flesh tissues from parental lines

| Class | Index | Compounds | PI 282448_25 DAP | M4-135_25 DAP |
| --- | --- | --- | --- | --- |
| Auxin | IA | 3-Indoleacrylic acid | 0.40±0.05^a^ | N/A |
|  | IAA-Ala | N-(3-Indolylacetyl)-L-alanine | 0.54±0.02^a^ | N/A |
|  | TRP | L-tryptophan | 19877.15±4127.34^a^ | 8638.03±1370.83^b^ |
|  | TRA | Tryptamine | 2.85±0.52^b^ | 7.50±0.69^a^ |
|  | IAA | Indole-3-acetic acid | 1.28±0.18^a^ | 0.63±0.04^b^ |
| CK | IP | N6-isopentenyladenine | 0.02±0.00^b^ | 0.03±0.01^a^ |
|  | cZR | cis-Zeatin riboside | 0.24±0.03^a^ | 0.12±0.01^b^ |
|  | oT | ortho-Topolin | 0.03±0.01^a^ | 0.01±0.01^b^ |
|  | tZOG | trans-Zeatin-O-glucoside | 1.97±0.08^a^ | 0.27±0.06^b^ |
|  | K9G | Kinetin-9-glucoside | 0.39±0.06^a^ | N/A |
|  | cZROG | cis-Zeatin-O-glucoside riboside | 0.34±0.03^a^ | N/A |
|  | DZ | Dihydrozeatin | 0.29±0.07^a^ | N/A |
|  | 2MeScZR | 2-Methylthio-cis-zeatin riboside | N/A | 0.02±0.01^a^ |
| GA | GA19 | Gibberellin A19 | 1.97±0.15^a^ | N/A |
|  | GA24 | Gibberellin A24 | 3.69±0.50^a^ | 0.50±0.08^b^ |
|  | GA15 | Gibberellin A15 | 1.55±0.59^b^ | 5.84±0.56^a^ |
|  | GA53 | Gibberellin A53 | 0.37±0.17^a^ | N/A |

Note: The difference of different letters was statistically significant (P<0.05); The unit of endogenous hormone content is ng/g. N/A indicates no detection.

**Table S2** QTL analysis of FD and FW in F_2_ population in the year of 2021 and 2022

| Trait | Year | Population | Chr | Left marker | Right marker | R^2^ (%) | LOD value |
| --- | --- | --- | --- | --- | --- | --- | --- |
| FD | 2021 | F_2_ (618) | 4 | *Chr04_12275277* | *Chr04_14591024* | 10.33 | 14.62 |
|  | 2022 | F_2_ (413) | 4 | *Chr04_13981204* | *Chr04_14771974* | 7.79 | 7.28 |
| FW | 2021 | F_2_ (618) | 4 | *Chr04_13981204* | *Chr04_14591024* | 8.33 | 3.55 |
|  | 2022 | F_2_ (413) | 4 | *Chr04_13289189* | *Chr04_14771974* | 6.69 | 4.21 |

**Table S3** The KASP primers information for background selection

| Primer name | SNP | Sequences （5’→3’） |  | |
| --- | --- | --- | --- | --- |
| *Chr01_5042606* | A/G | X: GAAGGTGACCAAGTTCATGCTTAGAGTACACCGAAATCACGGTATA |  | |
|  |  | Y: GAAGGTCGGAGTCAACGGATTGAGTACACCGAAATCACGGTATG |  | |
|  |  | C: GCAACATACTTCTTGTAGCTTGCATTATC |  | |
| *Chr01_10025793* | G/T | X: GAAGGTGACCAAGTTCATGCTTAAGCAACAGAGAACATTCGATAGAC |  | |
|  |  | Y: GAAGGTCGGAGTCAACGGATTCTAAGCAACAGAGAACATTCGATAGAA |  | |
|  |  | C: GCATCGATTTTGGTCAGATTTGACATTTTG |  | |
| *Chr01_15046612* | T/G | X: GAAGGTGACCAAGTTCATGCTGCACTCTCTTTCCATCTACTCTCTA |  | |
|  |  | Y: GAAGGTCGGAGTCAACGGATTCACTCTCTTTCCATCTACTCTCTC |  | |
|  |  | C: GTATGGAGTGGGAAGTAGGGGAG |  | |
| *Chr01_20039850* | A/C | X: GAAGGTGACCAAGTTCATGCTACTGCAGACGAAACGACATGGA |  | |
|  |  | Y: GAAGGTCGGAGTCAACGGATTCTGCAGACGAAACGACATGGC |  | |
|  |  | C: AGTTACGCTCTCCTTCGTGTCAAAC |  | |
| *Chr01_25221375* | G/A | X: GAAGGTGACCAAGTTCATGCTTATACACAATCACATTATTTACTCGCC |  | |
|  |  | Y: GAAGGTCGGAGTCAACGGATTTCTATACACAATCACATTATTTACTCGCT |  | |
|  |  | C: TACCATGGGCAGCTGTGTAGTCATT |  | |
| *Chr01_30918552* | G/A | X: GAAGGTGACCAAGTTCATGCTCATACTCAAAGCACAAGCAGATAATTC |  | |
|  |  | Y: GAAGGTCGGAGTCAACGGATTCATACTCAAAGCACAAGCAGATAATTT |  | |
|  |  | C: CTAATGATCATTGTTATTTTCTTTCACCTTAATG |  | |
| *Chr01_35922021* | T/C | X: GAAGGTGACCAAGTTCATGCTACACCACATTTGAAGGCGTGTTTTAT |  | |
|  |  | Y: GAAGGTCGGAGTCAACGGATTCACCACATTTGAAGGCGTGTTTTAC |  | |
|  |  | C: TCAAAGGTCAGAAGAAAATTGCAGTGAC |  | |
| *Chr02_5617445* | A/C | X: GAAGGTGACCAAGTTCATGCTTGTTAGTGAGATAAAACTTGTATTAATAGGT |  | |
|  |  | Y: GAAGGTCGGAGTCAACGGATTGTTAGTGAGATAAAACTTGTATTAATAGGG |  | |
|  |  | C: CGAATTGTGTTTCAAAGTGGTTTGAAATTAC |  | |
| *Chr02_10505147* | G/A | X: GAAGGTGACCAAGTTCATGCTGTGGCTAGAGGTATGTCTTCGC |  | |
|  |  | Y: GAAGGTCGGAGTCAACGGATTTGTGGCTAGAGGTATGTCTTCGT |  | |
|  |  | C: CCACTCAAAGGAAAAGAAAAACTCAAATTAG |  | |
| *Chr02_15543647* | A/G | X: GAAGGTGACCAAGTTCATGCTTCTGAGCACCATGACCGAATTGA |  | |
|  |  | Y: GAAGGTCGGAGTCAACGGATTCTGAGCACCATGACCGAATTGG |  | |
|  |  | C: ACAAAAGAATTGGGTCCAGCTGCTG |  | |
| *Chr02_20887769* | G/A | X: GAAGGTGACCAAGTTCATGCTGTACCTAGAAGTTAATATCCTACGTG |  | |
|  |  | Y: GAAGGTCGGAGTCAACGGATTATGTACCTAGAAGTTAATATCCTACGTA |  | |
|  |  | C: TGACCCCACAACGTTTGAGTGTCAA |  | |
| *Chr02_25870717* | C/T | X: GAAGGTGACCAAGTTCATGCTACACATGCCTTAGTCTCCTCAAC |  | |
|  |  | Y: GAAGGTCGGAGTCAACGGATTCACACATGCCTTAGTCTCCTCAAT |  | |
|  |  | C: GTGATGTGTTGATAAGAAGAATAGTAAAATGC |  | |
| *Chr03_10686913* | G/C | X: GAAGGTGACCAAGTTCATGCTGAAATGATGCCCTAGCTGTTTACAG |  | |
|  |  | Y: GAAGGTCGGAGTCAACGGATTGAAATGATGCCCTAGCTGTTTACAC |  | |
|  |  | C: CTGATTGCCATGGATCGAGCTTTATG |  | |
| *Chr03_15581698* | T/C | X: GAAGGTGACCAAGTTCATGCTCCTCCGATGACGAACTCGGT |  | |
|  |  | Y: GAAGGTCGGAGTCAACGGATTCCTCCGATGACGAACTCGGC |  | |
|  |  | C: TGGCTCGTGGAGGCGTCGGA |  | |
| *Chr03_20658642* | A/G | X: GAAGGTGACCAAGTTCATGCTGGCAAAGGCCTTGCTTGGTCAA |  | |
|  |  | Y: GAAGGTCGGAGTCAACGGATTGCAAAGGCCTTGCTTGGTCAG |  | |
|  |  | C: GTGAAAGGTTTGCCTCTCCTCTTTAG |  | |
| *Chr03_25657292* | G/A | X: GAAGGTGACCAAGTTCATGCTTACACTTGAGTTTCTCAAAACAAAGAAG |  | |
|  |  | Y: GAAGGTCGGAGTCAACGGATTGTACACTTGAGTTTCTCAAAACAAAGAAA | |  |
|  |  | C: AATATCTCGCCCAGTTTCACAATTTAATTTC | |  |
| *Chr03_30815565* | C/G | X: GAAGGTGACCAAGTTCATGCTCACTTCTGTTTGTGTCTCAGTGATG | |  |
|  |  | Y: GAAGGTCGGAGTCAACGGATTCACTTCTGTTTGTGTCTCAGTGATC | |  |
|  |  | C: GAACCATCAACCAGGGAAGTCCG | |  |
| *Chr04_5047046* | C/A | X: GAAGGTGACCAAGTTCATGCTCTACCTCTTTTGGAACTAGACTCC | |  |
|  |  | Y: GAAGGTCGGAGTCAACGGATTATCTACCTCTTTTGGAACTAGACTCA | |  |
|  |  | C: ATGATGGCCCTCAGAGTGATGATAG | |  |
| *Chr04_10146033* | A/T | X: GAAGGTGACCAAGTTCATGCTCCTCAGTGCCCCTTTCAAGCTT | |  |
|  |  | Y: GAAGGTCGGAGTCAACGGATTCCTCAGTGCCCCTTTCAAGCTA | |  |
|  |  | C: TGTGAGCTACGATTAGGGTGAGTTG | |  |
| *Chr04_15142103* | C/G | X: GAAGGTGACCAAGTTCATGCTTCTCAATACAATATGCTTAGTCTCCG | |  |
|  |  | Y: GAAGGTCGGAGTCAACGGATTTCTCAATACAATATGCTTAGTCTCCC | |  |
|  |  | C: AATATAGCGAATGGGAGGGGATCTC | |  |
| *Chr04_17013131* | G/A | X: GAAGGTGACCAAGTTCATGCTGATATGCTTCCTATCTCTCTCTATG | |  |
|  |  | Y: GAAGGTCGGAGTCAACGGATTCGATATGCTTCCTATCTCTCTCTATA | |  |
|  |  | C: AACAGAGTTGTGGGACTCAATTTTTGTG | |  |
| *Chr04_17472681* | T/A | X: GAAGGTGACCAAGTTCATGCTTTCACATTGTTCTTGCTACATCTTACT | |  |
|  |  | Y: GAAGGTCGGAGTCAACGGATTTTCACATTGTTCTTGCTACATCTTACA | |  |
|  |  | C: GTACAATTTTCCTCTTGAAAACTTATAAACGATC | |  |
| *Chr04_20446566* | T/C | X: GAAGGTGACCAAGTTCATGCTTGATATGCAACTTCAAGTGTTGAATAGTT | |  |
|  |  | Y: GAAGGTCGGAGTCAACGGATTATATGCAACTTCAAGTGTTGAATAGTC | |  |
|  |  | C: GGTTACATAGAAAAGCAATTAAGTACTTGTTTC | |  |
| *Chr04_25808258* | C/T | X: GAAGGTGACCAAGTTCATGCTCAACTCCGTGAAACAAAACAGTGAC | |  |
|  |  | Y: GAAGGTCGGAGTCAACGGATTACAACTCCGTGAAACAAAACAGTGAT | |  |
|  |  | C: TAATAGCCAATGAATGGATGATGAATCAATG | |  |
| *Chr04_30890256* | C/A | X: GAAGGTGACCAAGTTCATGCTCCTTAAATGTTATACCACTTGGGCC | |  |
|  |  | Y: GAAGGTCGGAGTCAACGGATTCCTTAAATGTTATACCACTTGGGCA | |  |
|  |  | C: TCAATGAAACTATTTCCTATTGAGGGACAAA | |  |
| *Chr05_5379865* | G/C | X: GAAGGTGACCAAGTTCATGCTTCGAATTTGAAGGTGCATTTTTAGCG | |  |
|  |  | Y: GAAGGTCGGAGTCAACGGATTTCGAATTTGAAGGTGCATTTTTAGCC | | |
|  |  | C: GCAAACGCTACCACTTGCAAGAATG | | |
| *Chr05_10206710* | A/G | X: GAAGGTGACCAAGTTCATGCTTTCCCAAGTGATTAGGCATTAAGGT | | |
|  |  | Y: GAAGGTCGGAGTCAACGGATTCCCAAGTGATTAGGCATTAAGGC | | |
|  |  | C: TTCCTCGCGTTGCAACACATCTTAC | | |
| *Chr05_15888901* | A/C | X: GAAGGTGACCAAGTTCATGCTAGTTCACAAGAGGGAAGGGGAAA | | |
|  |  | Y: GAAGGTCGGAGTCAACGGATTGTTCACAAGAGGGAAGGGGAAC | | |
|  |  | C: TCATATTACAGCAAGCAACATTCCAAGC | | |
| *Chr05_20355855* | A/G | X: GAAGGTGACCAAGTTCATGCTAACAGAAACTGATTATTGTTGTGGTTGA | | |
|  |  | Y: GAAGGTCGGAGTCAACGGATTCAGAAACTGATTATTGTTGTGGTTGG | | |
|  |  | C: AGCTACTGATAAAAGGAAGAATGTGGTG | | |
| *Chr06_10074915* | T/A | X: GAAGGTGACCAAGTTCATGCTGAGCTCGCTGCCCTTTAATAACT | | |
|  |  | Y: GAAGGTCGGAGTCAACGGATTGAGCTCGCTGCCCTTTAATAACA | | |
|  |  | C: CATTTATTACTGCACACCACCTGTTGG | | |
| *Chr06_20460803* | A/T | X: GAAGGTGACCAAGTTCATGCTAAAATGTAACCAATATGAGTAATCAACACT | | |
|  |  | Y: GAAGGTCGGAGTCAACGGATTAAAATGTAACCAATATGAGTAATCAACACA | | |
|  |  | C: TTTGTTCTTATTGTTGCCACTTTTTGATGTG | | |
| *Chr06_25186833* | A/C | X: GAAGGTGACCAAGTTCATGCTCTTCGGGCTATTTTACGTAAGCGAT | | |
|  |  | Y: GAAGGTCGGAGTCAACGGATTTCGGGCTATTTTACGTAAGCGAG | | |
|  |  | C: ACGTTCATCCGCACATGATGCTTCA | | |
| *Chr06_31092699* | G/A | X: GAAGGTGACCAAGTTCATGCTATTCTTCTCAGAAAACTGCTCGTAG | | |
|  |  | Y: GAAGGTCGGAGTCAACGGATTGATTCTTCTCAGAAAACTGCTCGTAA | | |
|  |  | C: TGTTTTCTTGTTTTACAAAGAAACTCCATTTTTC | | |
| *Chr06_36655657* | A/G | X: GAAGGTGACCAAGTTCATGCTCCTGCATGATCAGATAGCAACCA | | |
|  |  | Y: GAAGGTCGGAGTCAACGGATTCTGCATGATCAGATAGCAACCG | | |
|  |  | C: GATTATGGTTATGCAAGAAACTAAGTACTAAC | | |
| *Chr07_5051385* | C/T | X: GAAGGTGACCAAGTTCATGCTCATCTGAACACCCAGCATAAGAC | | |
|  |  | Y: GAAGGTCGGAGTCAACGGATTCCATCTGAACACCCAGCATAAGAT | | |
|  |  | C: ACGAATGTACAACCATTTTTCACTTTTGATC | | |
| *Chr07_10458389* | A/T | X: GAAGGTGACCAAGTTCATGCTACCACAAAAGCGAAGACTAGTTTTGA | | |
|  |  | Y: GAAGGTCGGAGTCAACGGATTACCACAAAAGCGAAGACTAGTTTTGT | | |
|  |  | C: CGTTACTAATCTTACTAAGCATTCTTGAATTTTG | | |
| *Chr07_15158780* | T/A | X: GAAGGTGACCAAGTTCATGCTCATAACCTCCCCAGATAGAGTAGT | | |
|  |  | Y: GAAGGTCGGAGTCAACGGATTCATAACCTCCCCAGATAGAGTAGA | | |
|  |  | C: GAAGTAGAACCATTGAGTGGTGTTTTATC | | |
| *Chr07_20174385* | T/C | X: GAAGGTGACCAAGTTCATGCTGCCAGAAAGTCATAAATTGTCCACAAT | | |
|  |  | Y: GAAGGTCGGAGTCAACGGATTCCAGAAAGTCATAAATTGTCCACAAC | | |
|  |  | C: CGCTTATCAGACAATTCTTTCAGTTGTATG | | |
| *Chr07_25291843* | T/A | X: GAAGGTGACCAAGTTCATGCTCAGATACTACGGTTTCAGTTACAGTAT | | |
|  |  | Y: GAAGGTCGGAGTCAACGGATTCAGATACTACGGTTTCAGTTACAGTAA | | |
|  |  | C: CCCATCCATGTTTCTACTGTGGAAAC | | |
| *Chr08_5212408* | C/A | X: GAAGGTGACCAAGTTCATGCTCTACAGTTCTCACTACCGATACG | | |
|  |  | Y: GAAGGTCGGAGTCAACGGATTAACTACAGTTCTCACTACCGATACT | | |
|  |  | C: CAGTAAATTCCTGAACCATGGCTCG | | |
| *Chr08_10361981* | A/G | X: GAAGGTGACCAAGTTCATGCTGTGTAAAGCTGAAGCTGTCACCA | | |
|  |  | Y: GAAGGTCGGAGTCAACGGATTTGTAAAGCTGAAGCTGTCACCG | | |
|  |  | C: ACAACTCTCTTCCCTTCCAACACTG | | |
| *Chr08_15313382* | A/G | X: GAAGGTGACCAAGTTCATGCTTTGTTTACATCACTCTTGTCAAAAAGACA | | |
|  |  | Y: GAAGGTCGGAGTCAACGGATTGTTTACATCACTCTTGTCAAAAAGACG | | |
|  |  | C: GTAACAAGCATCAAAACCTGTACATTGATG | | |
| *Chr08_20009426* | C/T | X: GAAGGTGACCAAGTTCATGCTGATCGCTACTATAATGTGTTGATTCG | | |
|  |  | Y: GAAGGTCGGAGTCAACGGATTAAGATCGCTACTATAATGTGTTGATTCA | | |
|  |  | C: GATATGTTTGGAAGGTGGCTACCATG | | |
| *Chr08_25162317* | T/G | X: GAAGGTGACCAAGTTCATGCTAAACCAAAGTCCATAGTAGCACCAT | | |
|  |  | Y: GAAGGTCGGAGTCAACGGATTACCAAAGTCCATAGTAGCACCAG | | |
|  |  | C: CTCTCACTCTTAGGACGTTGATTTATTTG | | |
| *Chr08_30009623* | A/G | X: GAAGGTGACCAAGTTCATGCTAAATCTAAACGATCGTTTACCAAACAATAA | | |
|  |  | Y: GAAGGTCGGAGTCAACGGATTAATCTAAACGATCGTTTACCAAACAATAG | | |
|  |  | C: ACAACGCACGTAATATATTTGATATACGATC | | |
| *Chr09_5054766* | G/T | X: GAAGGTGACCAAGTTCATGCTGCATGCTAGGGTAAGGATGCG | | |
|  |  | Y: GAAGGTCGGAGTCAACGGATTTGCATGCTAGGGTAAGGATGCT | | |
|  |  | C: TCACCACTAATCAATAAATCAGATGACTATC | | |
| *Chr09_15000924* | A/G | X: GAAGGTGACCAAGTTCATGCTTTTCCCCTAACTTGTAAAGTCAATCTT | | |
|  |  | Y: GAAGGTCGGAGTCAACGGATTTTCCCCTAACTTGTAAAGTCAATCTC | | |
|  |  | C: CACTGTACAATATGAGCACATACATTACAAA | | |
| *Chr09_20118729* | A/T | X: GAAGGTGACCAAGTTCATGCTATTATCGCGGTGTCGCTTGCAAA | | |
|  |  | Y: GAAGGTCGGAGTCAACGGATTATTATCGCGGTGTCGCTTGCAAT | | |
|  |  | C: AACTTCCCAAGTGCAACCATTGTAATAG | | |
| *Chr09_25146323* | T/C | X: GAAGGTGACCAAGTTCATGCTCAATCAGGTAAAAGGATATCAACATCAA | | |
|  |  | Y: GAAGGTCGGAGTCAACGGATTAATCAGGTAAAAGGATATCAACATCAG | | |
|  |  | C: GCACACAATTTTAGTGAAGAATGAAATCAAAC | | |
| *Chr10_5032152* | C/T | X: GAAGGTGACCAAGTTCATGCTGGACAAAGGTATCCCATAATACCC | | |
|  |  | Y: GAAGGTCGGAGTCAACGGATTGGGACAAAGGTATCCCATAATACCT | | |
|  |  | C: GTTGGCTTTCCGGGGATGGGTTA | | |
| *Chr10_10491740* | C/T | X: GAAGGTGACCAAGTTCATGCTGAGATGAATGAGGTACAACAGGTC | | |
|  |  | Y: GAAGGTCGGAGTCAACGGATTAAGAGATGAATGAGGTACAACAGGTT | | |
|  |  | C: CTTTCCCCCTGCCTTTAAGCATTTG | | |
| *Chr10_15292154* | T/A | X: GAAGGTGACCAAGTTCATGCTGGGCATTTTCAGGATTTGCTTATCTA | | |
|  |  | Y: GAAGGTCGGAGTCAACGGATTGGGCATTTTCAGGATTTGCTTATCTT | | |
|  |  | C: CACCATTAAGACAGACTTATGGAAACTG | | |
| *Chr10_20261784* | G/T | X: GAAGGTGACCAAGTTCATGCTTTCTTCTGGAGGTTTTGCTGCTG | | |
|  |  | Y: GAAGGTCGGAGTCAACGGATTGTTTCTTCTGGAGGTTTTGCTGCTT | | |
|  |  | C: CTTGTATAAAAGCCAATTCCATATTGCAATC | | |
| *Chr10_25317618* | C/G | X: GAAGGTGACCAAGTTCATGCTAAGGTTTTATGTCAAAGAAGGAAAAGTTC | | |
|  |  | Y: GAAGGTCGGAGTCAACGGATTAAGGTTTTATGTCAAAGAAGGAAAAGTTG | | |
|  |  | C: ACACCTAGTATTGTCTCTTTTGATACTTAAC | | |
| *Chr11_5189122* | A/T | X: GAAGGTGACCAAGTTCATGCTTTTGAAAAGAACTTGTGAGTCAAACTCAT | | |
|  |  | Y: GAAGGTCGGAGTCAACGGATTTTTGAAAAGAACTTGTGAGTCAAACTCAA | | |
|  |  | C: GGCCTGATTACTATGAAATCGAAGAAAAC | | |
| *Chr11_10364250* | A/T | X: GAAGGTGACCAAGTTCATGCTCTGTTTGGTCTTCTACAAACACAACAA | | |
|  |  | Y: GAAGGTCGGAGTCAACGGATTCTGTTTGGTCTTCTACAAACACAACAT | | |
|  |  | C: ACGATCAAGTTCAGAAGAAGCTCATAAG | | |
| *Chr11_15121814* | A/T | X: GAAGGTGACCAAGTTCATGCTAGGTCATATCTTGCTTTCCATCTCAA | | |
|  |  | Y: GAAGGTCGGAGTCAACGGATTAGGTCATATCTTGCTTTCCATCTCAT | | |
|  |  | C: AGTGTTTATAAAGCAGAGACGTTCCTTG | | |
| *Chr11_20402436* | A/T | X: GAAGGTGACCAAGTTCATGCTGACCTCTTGGACTACACTTCTTTGT | | |
|  |  | Y: GAAGGTCGGAGTCAACGGATTGACCTCTTGGACTACACTTCTTTGA | | |
|  |  | C: CTTCAACTTTTGAGTCATTCGATAATTTAAAATG | | |
| *Chr11_25414060* | C/T | X: GAAGGTGACCAAGTTCATGCTTCAGATCACTGAGAAAGGAAAACAAC | | |
|  |  | Y: GAAGGTCGGAGTCAACGGATTATTCAGATCACTGAGAAAGGAAAACAAT | | |
|  |  | C: AGTTTCCTTTAAACATGCTGAATATATTCTATTG | | |
| *Chr11_30940986* | T/A | X: GAAGGTGACCAAGTTCATGCTGATACCACACTTAGCCTCTGATCT | | |
|  |  | Y: GAAGGTCGGAGTCAACGGATTGATACCACACTTAGCCTCTGATCA | | |
|  |  | C: AAGATTCTACCAGGATACGAGATTGATC | | |
| *Chr12_5226635* | A/G | X: GAAGGTGACCAAGTTCATGCTTCCAAGGAATCATGAGACCTCCT | | |
|  |  | Y: GAAGGTCGGAGTCAACGGATTCCAAGGAATCATGAGACCTCCC | | |
|  |  | C: AGAGATGGTGAGTAGGAGAACGTTC | | |
| *Chr12_10110225* | A/G | X: GAAGGTGACCAAGTTCATGCTCGGTAGAAGAAGAAGAAGGGTCA | | |
|  |  | Y: GAAGGTCGGAGTCAACGGATTGGTAGAAGAAGAAGAAGGGTCG | | |
|  |  | C: AGCCAATCCCAACAACTTCTCTCTC | | |
| *Chr12_15099994* | C/G | X: GAAGGTGACCAAGTTCATGCTCACGAACATAAGCACAAGAAGCAC | | |
|  |  | Y: GAAGGTCGGAGTCAACGGATTCACGAACATAAGCACAAGAAGCAG | | |
|  |  | C: GGTTGTCCTTAAAAATGCTGAATACGAC | | |
| *Chr12_20326841* | A/G | X: GAAGGTGACCAAGTTCATGCTCAGATGCCTGAAAATAAACATCACACA | | |
|  |  | Y: GAAGGTCGGAGTCAACGGATTAGATGCCTGAAAATAAACATCACACG | | |
|  |  | C: GTCCGACTGATTCTTGATTTTATGAGTTC | | |
| *Chr12_25069233* | A/G | X: GAAGGTGACCAAGTTCATGCTAGGAAAAGAGTCGAGTCCATGGA | | |
|  |  | Y: GAAGGTCGGAGTCAACGGATTGGAAAAGAGTCGAGTCCATGGG | | |
|  |  | C: CCAAATCTAAACAAATCTTTTCAAAGCAGATATC | | |

**Table S4** Candidate genes in the *Cmqft4.1* fine mapping region

| Gene ID | Physical distance | CDS(nsSNPs) | PRO | Gene annotation |
| --- | --- | --- | --- | --- |
| *MELO3C012889.2* | 13,975,265-13,977,985 | SNP^442th^(G→C, Val→Leu)  SNP^835th^(T→C, Ser→Pro)  SNP^1268th^(G→A, Arg→Lys)  SNP^1336th^(C→A, Gln→Lys) | SNP^433th^ (T→A, leading to change of TATA-box)  SNP^1216th^ (G→A, leading to change of CAAT-box) | 11S globulin seed storage protein 2-like |
| *MELO3C012890.2* | 14,008,038-14,010,285 | 0 | SNP^442th; 949th^ (C→A; A→C, leading to change of CAAT-box)  SNP^1013th^ (C→T, leading to change of Unnamed__1)  SNP^1106th^ (A→T, leading to change of TATA-box)  SNP^1156th^ (A→C, leading to change of W box) | Myb-related transcription factor |
| *MELO3C030522.2* | 14,022,808-14,025,981 | SNP^82th^(G→A, Val→Ile)  SNP^125th^(G→A, Cys→Tyr)  SNP^173th^(C→T, Ser→Phe) | SNP^284th^ (C→T, leading to change of W box)  SNP^377th^ (C→G, leading to change of CTAG-motif)  SNP^521th^ (T→C, leading to change of AACA_motif)  SNP^927th;978th^ (C→T;C→G, leading to change of Unnamed__2)  SNP^1582th^ (T→C, leading to change of Unnamed__4) | Unknown protein |
| *MELO3C030521.2* | 14,051,255-14,053,530 | SNP^113th^(T→A, Met→Lys) | SNP^1184th; 1272th; 1810th^ (G→A; T→A; G→A, leading to change of CAAT-box)  SNP^1313th^ (C→T, leading to change of GA-motif)  SNP^1884th^ (A→C, leading to change of TGACG-motif) | Cysteine synthase |
| *MELO3C012891.2* | 14,085,386-14,085,565 | SNP^17th^(C→T, Ser→Leu)  SNP^49th^(G→A, Val→Ile)  SNP^52th^(A→G, Lys→Glu) | SNP^387th^ (C→T, leading to change of Unnamed__4)  SNP^612th; 1421th^ (A→G; C→A, leading to change of CAAT-box)  SNP^631th^ (A→G, leading to change of TATA-box)  SNP^1326th^ (T→C, leading to change of G-Box) | Unknown protein |
| *MELO3C030785.2* | 14,085,611-14,087,389 | 0 | SNP^252th^ (C→T, leading to change of TCCC-motif)  SNP^336th^ (T→C, leading to change of TATC-box)  SNP^451th; 906th^ (C→T; A→T, leading to change of CAAT-box) | zinc finger BED domain-containing protein RICESLEEPER 2-like |
| *MELO3C012892.2* | 14,088,850-14,089,580 | SNP^133th^(G→A, Ala→Thr)  SNP^182th^(G→A, Arg→Lys)  SNP^239th^(C→T, Thr→Met) | SNP^1150th^ (A→C, leading to change of Myb)  SNP^1576th^ (C→G, leading to change of Unnamed__4) | Unknown protein |
| *MELO3C030527.2* | 14,113,603-14,113,911 | SNP^13th^(G→A, Ala→Thr)  SNP^127th^(G→A, Asp→Asn)  SNP^133th^(A→G, Arg→Gly)  SNP^160th^(G→A, Val→Met) | SNP^609th^ (C→A, leading to change of WRE3) | GATA zinc finger domain-containing protein 10-like isoform X2 |
| *MELO3C012895.2* | 14,124,547-14,124,732 | 0 | SNP^242th^ (T→C, leading to change of TATA-box)  SNP^278th; 282th; 577th; 902th;1712th^ (G→A; T→C; G→A; C→A; G→A, leading to change of CAAT-box) | inorganic phosphate transporter 1-4 |
| *MELO3C012896.2* | 14,164,520-14,165,518 | SNP^460th^(G→A, Lys→Glu)  SNP^543th^(3 bp insert, leading to a new amino acid form of Ala) | SNP^55th; 245th; 1033th^ (T→A, C→T, C→T, leading to change of TATA-box)  SNP^1062th^(286 bp deletion, contained ERE, STRE, TATA-box and CAAT- box)  SNP^1376th^(18 bp insert, contained a new WUN-motif) | ethylene-responsive transcription factor 1B-like |

**Table S5** The Phenotypic evaluation information of the 27 melon natural panel

| NO. | Accessions | Subspecies | Type | Flesh type | Flesh thickness (cm) |
| --- | --- | --- | --- | --- | --- |
| 1 | X151 | *melo* | cultivar | thick | 4.73 |
| 2 | X084 | *melo* | cultivar | thick | 5.50 |
| 3 | M4-135 | *melo* | cultivar | thick | 7.60 |
| 4 | M4-83 | *melo* | cultivar | thick | 6.88 |
| 5 | M4-134 | *melo* | cultivar | thick | 5.75 |
| 6 | M4-116 | *melo* | cultivar | thick | 5.11 |
| 7 | M4-5 | *melo* | cultivar | thick | 6.15 |
| 8 | MR-1 | *melo* | cultivar | thick | 4.74 |
| 9 | X147 | *melo* | cultivar | thick | 5.88 |
| 10 | M1-38 | *agrestis* | cultivar | thin | 3.60 |
| 11 | M1-39 | *agrestis* | cultivar | thin | 2.80 |
| 12 | M1-111 | *agrestis* | cultivar | thin | 3.12 |
| 13 | M1-113 | *agrestis* | cultivar | thin | 3.32 |
| 14 | M1-15 | *agrestis* | cultivar | thin | 3.36 |
| 15 | M1-96 | *agrestis* | cultivar | thin | 2.82 |
| 16 | X123 | *agrestis* | cultivar | thin | 3.50 |
| 17 | X060 | *agrestis* | cultivar | thin | 4.38 |
| 18 | X122 | *agrestis* | cultivar | thin | 3.60 |
| 19 | M1-7 | *agrestis* | cultivar | thin | 3.91 |
| 20 | ZTG01233 | *agrestis* | wild | thin | 0.60 |
| 21 | ZTG01244 | *agrestis* | wild | thin | 0.87 |
| 22 | ZTG01246 | *agrestis* | wild | thin | 0.59 |
| 23 | ZTG01251 | *agrestis* | wild | thin | 0.90 |
| 24 | ZTG01284 | *agrestis* | wild | thin | 0.72 |
| 25 | ZTG01285 | *agrestis* | wild | thin | 0.96 |
| 26 | PI 614174 | *agrestis* | wild | thin | 0.70 |
